# Attitudes to Noise Inside Dwellings in Three Megacities: Seoul, London, and São Paulo

**DOI:** 10.3390/ijerph17166005

**Published:** 2020-08-18

**Authors:** Pyoung Jik Lee, Carl Hopkins, Rafaella Penedo

**Affiliations:** Acoustics Research Unit, School of Architecture, University of Liverpool, Liverpool L69 7ZN, UK; carlh@liverpool.ac.uk (C.H.); rafapenedo@yahoo.com.br (R.P.)

**Keywords:** neighbour noise, outdoor noise, attitude, megacities

## Abstract

This study investigated people’s attitudes towards noise inside their homes. Online questionnaire surveys were conducted in Seoul, London, and São Paulo. The questionnaire was designed to assess annoyance caused by noise from neighbours and environmental noise (transportation). Information was also collected on situational, personal, and socio-demographic variables. Respondents that were more annoyed by outdoor noise inside their dwelling reported higher neighbour noise annoyance. In Seoul, neighbour noise was found to be more annoying than outdoor noise, and those with higher noise sensitivity reported higher annoyance towards neighbour noise. However, neighbour noise and outdoor noise was found to be equally annoying in London and São Paulo. For neighbour noise, the average percentage of respondents hearing structure-borne sources compared to airborne sources differed in each city. Most neighbour noise sources in São Paulo gave rise to higher annoyance ratings than Seoul and London. Education and income levels had a limited effect on annoyance and coping strategy. Annoyance with indoor noise from neighbours was found to have stronger relationships with cognitive and behavioural coping strategies than outdoor noise annoyance.

## 1. Introduction

More than half of the world’s population currently lives in urban areas and by 2050 it is expected that 68% of the world’s population will live in cities [1]. As global urbanisation continues, environmental pollution associated with urbanisation has become a social issue in many countries. According to the World Health Organization (WHO), traffic noise (e.g., road, rail, and air) has the second largest public health impact in Western Europe, behind only air pollution [2]. Thus, a number of studies [3,4,5] have examined the annoyance caused by traffic noise primarily in association with the noise level. In addition, attitudes towards environmental noise were investigated by considering the non-acoustic factors, such as personal and situational variables [6,7,8].

Inside buildings, occupants are exposed to environmental noise that is transmitted through the façade, as well as noise from activities in neighbouring flats and communal areas that is transmitted through the walls, floors, and doors. Langdon and Buller [9] reported that a quarter of the respondents heard noise from their neighbours. In fact, noise from neighbouring flats has been reported as the second most frequent noise source of annoyance in eight European cities, behind traffic noise [10]. About a quarter of the respondents reported sleep disturbance, which was attributable to the noise from traffic and neighbours. In particular, neighbour noise was the most frequent source of sleep disturbance in three cities. The proportions of respondents hearing neighbour noise between 1991 and 1999 increased from 19% to 25% in the UK [11]. An increase in the proportion of respondents hearing a specific noise source was found; specifically, people’s voices (11–17%), children (9–16%), radio/TV (9–12%), and doors banging (5–7%). The recent UK national noise attitude survey (2012) reported that 54% were bothered, annoyed, or disturbed to some extent by noise from neighbours and/or other people nearby [12]. The most frequent neighbour noise sources were voices/shouting/arguments, followed by dogs and radio/TV/music. Maschke and Niemann [13] reported that chronic exposure to severely annoying neighbour noise increased the health risk for children as well as the elderly. Lee et al. [14] identified stronger associations between the road traffic noise level and blood pressure for participants that reported higher indoor noise annoyance ratings. Recent studies also examined indoor noise as a concept of the soundscape [15,16,17]. Ma et al. [17] analysed the perceptual dimensions of indoor as well as outdoor sounds using semantic attributes and Torresin et al. [16] reviewed factors affecting indoor soundscapes in residential buildings based on 38 laboratory and field studies. This paper investigates annoyance towards neighbour noise and environmental noise when people are inside their homes. Sound insulation tends to be considered in terms of the protection provided against airborne or structure-borne sources of noise [18] and is one of the major criteria in building design [19]. In a UK noise attitude survey of residents in flats and houses [12], the majority of sources causing annoyance from neighbours were airborne sources, such as adults’ voices, parties held indoors, dogs, and telephones. The only reported structure-borne source was footsteps. Some studies on the impact sound from footsteps in flats have investigated psychophysiological responses to footstep noise through questionnaire surveys and laboratory experiments (e.g., [20,21,22,23]). A series of studies [24,25,26] have explored the perception of floor impact noise in flats by considering both acoustic and non-acoustic factors. A series of studies have reviewed the sound insulation performances of buildings obtained from field measurements [27] as well as the airborne and impact sound insulations from laboratory studies [28,29] in relation to subjective responses. Simmons [30] has suggested a question for assessing noise annoyance in buildings based on the International Standard [31]. However, a comparison of subjective responses to a range of airborne and structure-borne neighbour noise sources has not been carried out in different countries using the same questionnaire.

This study investigates three megacities: Seoul, London, and São Paulo, in Asia, Europe, and South America, respectively. Seoul and London have populations of more than 10 million and São Paulo is the fourth largest megacity in the world, with around 22 million inhabitants. The most common type of housing in megacities tends to be multi-story residential buildings. The Seoul Metropolitan Government [32] reported that flats accounted for over 62.8% of the housing units available in 2018. In London, 50% of the residents live in flats [33] whereas it is only 31.2% in São Paulo [34]. In Korea, more than 120,000 complaints about neighbour noise have been made since 2012 [35] and the majority of them were about noise from upstairs neighbours in flats. In particular, the number of complaints about footsteps from above has increased from 8795 in 2012 to 28,231 in 2018. In London, music from neighbours was among the top three noise complaints along with construction noise and commercial noise in 2016 [36]. All three cities follow the WHO environmental noise guidelines; however, environmental noise levels differ according to urban design and traffic volumes. As an indication of the environmental noise climate, the measured noise levels in Seoul ranged from 54 to 70 dB *L*_Aeq,16h_ (07:00 to 23:00) in 2018 [32], while London had predicted road traffic noise levels ranging from 55 to 86 dB *L*_Aeq,16h_ [37]. Official noise data from measurements or predictions are not available in São Paulo although there are measurements indicating that noise levels ranged from 51 to 80 dB *L*_Aeq,9h_ (08:00 to 17:00) in 2002 [38].

It was hypothesised that people’s attitudes to noise might be different across the three cities with different socio-economic conditions and acoustic environments. Thus, this study uses questionnaire surveys in three megacities to investigate the attitudes towards neighbour noise and outdoor transportation noise when people are inside their homes. It was also hypothesised that the situational and non-acoustic variables might affect noise annoyance and their effects might be different across the cities. In order to test this hypothesis, various situational and non-acoustic variables were assessed through the questionnaire.

## 2. Methods

### 2.1. Sample

Online questionnaire surveys were conducted in Seoul, London, and São Paulo in the Korean, British English, and Portuguese languages, respectively. For Seoul and São Paulo, the online surveys were designed using Google forms [39]. Using snowball sampling, the researchers initially sent a text message with a link to the survey to around 10 friends and asked them to send the link to their friends, colleagues, and family. Those who completed the survey were also asked to disseminate the link to their friends and relatives. Using this method, the survey collected around 100 responses from each city within three weeks. However, the participants were recruited in London through SmartSurvey [40], who have their own registered participant pool. Eligible participants were those who aged 18 years and above and lived in each city.

In Seoul, London, and São Paulo, the number of completed questionnaires were 117, 144, and 102, respectively (see Table 1). The survey in Korea was conducted in December 2015, while the surveys in London and São Paulo were performed in November 2017 and in January 2020, respectively. Of these 363 completed questionnaires, 174 (48.2%) were from male respondents and 188 (51.8%) from female respondents. The majority of respondents (75.4%) were aged between 18 and 50 years old with the remainder being above 50 years old. The majority of the respondents in all three cities were educated to university degree level and more than one-half were fulltime workers. Approximately 35% of the respondents lived with children, while the remainder lived alone, with other family members, or with other people. The type of dwelling in which the respondents lived differed between the cities. In Seoul, all respondents except two lived in flats. In London and São Paulo, 59.0% and 74.5% of the respondents lived in flats, respectively.

### 2.2. Questionnaire Design

The climate and ventilation strategies are different in the three cities. For example, the annual average temperature in São Paulo is higher than those in Seoul and London. The level of control over the indoor environment also affects the perceived quality of the indoor soundscape [41]. Thus, the questionnaire asked about the noise environment with the windows closed. First, the questionnaire asked whether a source of noise could be heard when the participant was in their bedroom, living room, or home office with the windows closed. If they responded positively, they were asked to rate their annoyance with that source. It was not possible to estimate (a) the environmental noise exposure on each façade of these rooms; (b) the sound insulation of the façade against the environmental noise source(s); or (c) the airborne and impact sound insulation that existed between the participants and each of their neighbours. The national building regulations that were in force at the time of construction provide an indication of the minimum requirement for airborne, impact, and façade sound insulation. However, compliance with these regulations is only known (if at all) for a fraction of the recently constructed dwellings, and it is rarely known for older dwellings. Hence this study focussed on noises that the participants heard.

The questionnaire is provided in the Supplementary Text Material S1 and was divided into four main sections. The first section concerned the respondents’ responses to four different types of transportation noise: (1) road traffic on major roads; (2) road traffic on minor roads; (3) airplanes and/or helicopters; and (4) trains and/or trams. Respondents were asked if they heard each of these four types and only those who responded positively were asked to assess the level of annoyance caused by the noise from that particular transportation source. Noise annoyance was rated using an 11-point scale (0 = “not at all” and 10 = “extremely”) as recommended by the ICBEN team [42] and the International Standard, ISO 15666 [31]. Participants were provided with the following instruction: “Thinking about the last 12 months or so, when you are in your home, how much does specific noise (e.g., noise from major roads) annoy you?”

In the second section, participants were asked if they heard noise from the neighbour’s dwelling when they were in their bedroom/living room/home office with the windows closed. Only participants who heard the noise were asked to rate the level of noise annoyance caused by neighbour noise using the same 11-point scale. If the respondents confirmed that they heard a specific noise source they were asked to rate their annoyance with that source. A total of ten major noise sources were selected based on previous studies [43,44,45,46] and these were classified as either structure-borne or airborne sources. Structure-borne sources were footsteps (including jumping and running), dropped objects, movement of furniture, door closing, home appliances (e.g., a washing machine, dishwasher, tumble dryer, and vacuum cleaner), and water installations (e.g., sounds from pipes, plumbing, flushing toilets, showers, and baths). Airborne sources were talking/shouting, TV/music, telephones ringing, and dogs barking.

In the third section, participants who lived in flats were asked if they heard talking/shouting, footsteps, doors closing, and service installations (e.g., lift, power generator, and ventilation machinery) from the communal areas in the block of flats. Those who responded positively were asked to rate the noise annoyance using the same 11-point scale.

The fourth section of the questionnaire was used to measure the situational and non-acoustic variables [47]. Participants were asked if they knew which of their neighbours caused the noise and were asked to rate their relationship with those neighbours. As a personal variable, noise sensitivity was measured using nine items from Weinstein’s noise sensitivity scale [48] using a five-point scale (0 = “disagree strongly” and 5 = “agree strongly”). Nine items were used to measure their reaction to noise in terms of a behavioural coping strategy; for example, participants were asked to indicate their agreement with the statement “I increase the volume of the radio/TV/music”. Cognitive and behavioural coping strategies were measured using a five-point scale (0 = “never” and 5 = “always”). Six items were used to measure the cognitive coping strategy; for example, participants were asked how often they react according to the statement “I accept that I cannot do anything to stop the noise”. Participants were also asked if, and how, they had complained about the noise from their neighbour’s property.

### 2.3. Data Analysis

Annoyance ratings were converted to the percentage of highly annoyed (%HA) with a cut-off at 72 on a scale from zero to 100. Responses from the 11-point scale were translated into a scale from 0 to 100, and it was assumed that the categories divide this scale into equally spaced intervals. The percentage of the responses above the cut-off were then calculated as the %HA. Statistical analysis was carried out using SPSS for Windows (Version 22.0, SPSS Inc., Chicago, IL, USA). One-way analysis of variance (one-way ANOVA) was used to compare the noise annoyance ratings between cities with paired samples *t*-tests to compare the annoyance ratings from indoor and outdoor noise. Independent samples *t*-tests were used to compare the responses between groups (e.g., the low and high noise-sensitivity groups). The Shapiro–Wilk normality test result indicated that several variables were not normally distributed; therefore, Spearman rank correlation coefficients were computed to examine correlations between the variables. Kruskal–Wallis and Mann–Whitney U tests (with Bonferroni correction) were used for the comparisons of several variables that were not normally distributed. A two-way interaction technique was used to examine the moderation effect of noise sensitivity on the relationship between the dependent variable (i.e., coping strategy) and independent variable (i.e., noise annoyance). Before the analysis, all the variables were mean centred by subtracting a variable’s mean from all the observations to avoid multicollinearity issues. Regression analyses were then performed, including the independent variable, moderator, and their interaction term (independent variable × moderator). The interaction effects were plotted using the unstandardised regression coefficients, means, and standard deviations of the independent variable and moderator [49]. In the present study, *p* values of less than 5% (*p* < 0.05) were considered as statistically significant.

## 3. Results and Discussion

### 3.1. Outdoor Noise Annoyance

**Figure 1:**
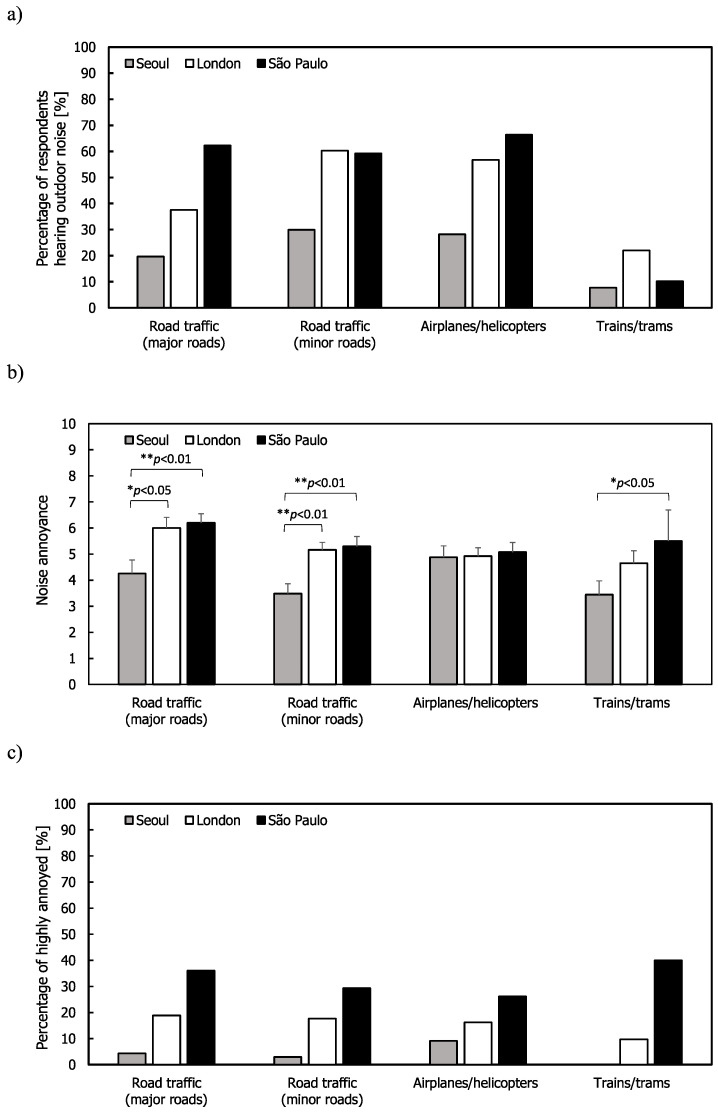
Outdoor noise: (**a**) Percentage of respondents hearing noise for all the respondents; (**b**) mean annoyance; and (**c**) percentage highly annoyed (%HA) ratings. Error bars indicate standard errors (* *p* < 0.05, ** *p* < 0.01).

For outdoor transportation noise sources, Figure 1 shows the mean annoyance and %HA ratings along with the percentage of respondents hearing these sources across the three cities. The percentage of respondents in Seoul indicating that they heard these sources was lower than in London and São Paulo. In Seoul, less than 30% of the respondents heard noise from road traffic and airplanes/helicopters and only 7.7% heard noise from trains/trams. The highest mean annoyance ratings occurred in London and São Paulo for road traffic on major roads. For road traffic on major and minor roads, the mean annoyance ratings were higher in London and São Paulo than in Seoul. This might be because of lower external noise levels and higher façade insulation in Seoul. For airplanes/helicopters, the mean annoyance ratings were similar in all three cities. For trains/trams, the mean annoyance rating and %HA in London and São Paulo were higher than in Seoul; however, the percentage of the respondents from the three cities was less than 25%. The highest percentage of respondents hearing road traffic (combination of major and minor roads) and airplanes/helicopters inside their dwellings came from São Paulo, although the percentages for road traffic from minor roads and airplanes/helicopters were similar in London.

For road traffic (major and minor roads), respondents from São Paulo had the highest mean annoyance ratings with Seoul having the lowest ratings. In terms of %HA, annoyance was lower in Seoul than the other two cities; this might be attributed to higher façade insulation because around 96% of windows in Korea were found to be double glazed in 2016 [50] (in the UK it was 85% in 2018 [51]) and noise reduction increased due to balconies being fitted with additional windows and doors. The availability of a quiet side of a home has been found to reduce noise annoyance [52]. One-way ANOVA indicated that the mean annoyance ratings were different between Seoul and São Paulo, except for airplanes/helicopters (F(2,140) = 6.456, *p* < 0.01 for road traffic from major road; F(2,177) = 8.666, *p* < 0.01 for road traffic from minor roads; F(2,51) = 3.374, *p* < 0.05 for trains/trams). Post hoc comparisons via Tukey’s test indicated that the mean annoyance ratings due to road traffic from major and minor roads in Seoul were statistically different from those in London and São Paulo. The difference in annoyance ratings of trains/trams between Seoul and São Paulo was also significant.

**Figure 2:**
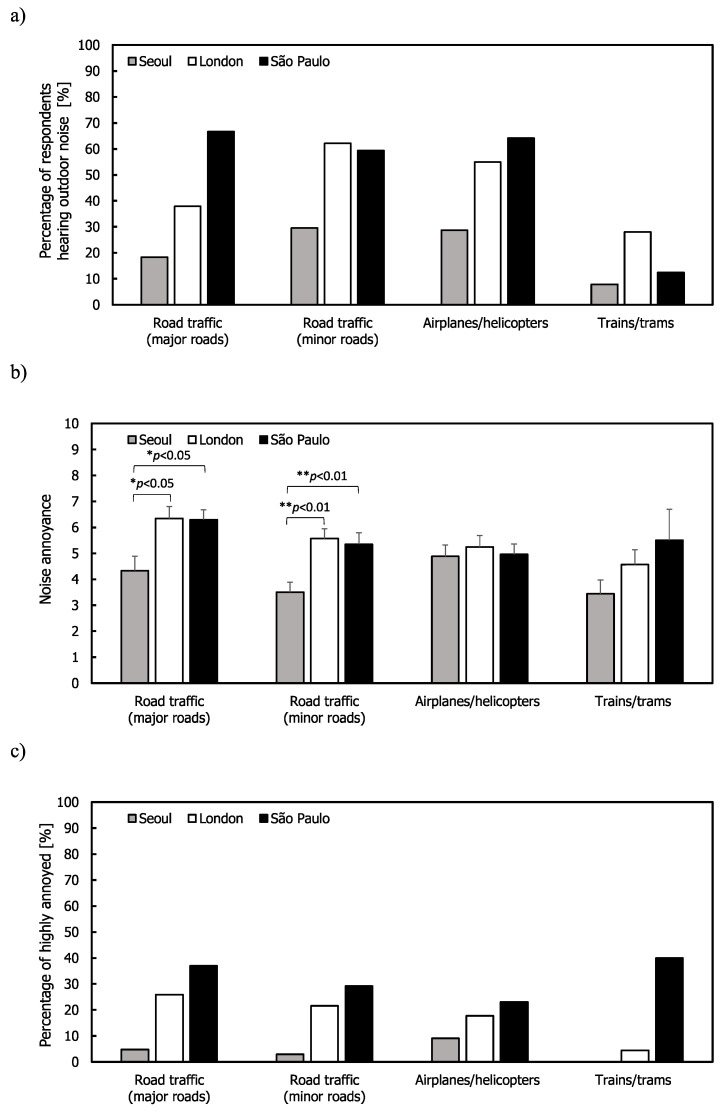
Outdoor noise: (**a**) Percentage of respondents hearing noise for the respondents living in flats; (**b**) mean annoyance; (**c**) %HA ratings. Error bars indicate standard errors (* *p* < 0.05, ** *p* < 0.01).

Figure 2 shows the results from the residents living in flats and indicates similar tendencies to the findings from all the residents living in different types of buildings. The percentages hearing noise in Seoul were lower than those in other cities and the trains/trams noise showed the lowest percentages for all the cities. Annoyance ratings of the road traffic noise on major roads were highest in London and São Paulo, while Seoul showed lower annoyance ratings than the other cities for all the outdoor noise sources. Results of the one-way ANOVA showed that the mean annoyance ratings were different across the cities for road traffic noise (F(2,104) = 4.858, *p* < 0.01 for major roads and F(2,130) = 6.892, *p* < 0.01 for minor roads). Post hoc comparisons via Tukey’s test also indicated that the mean annoyance ratings of road traffic from major and minor roads in Seoul were statistically lower than those in London and São Paulo.

In addition, the effect of the floor on which the respondents live (i.e., ground, intermediate, and top floors) on the annoyance of outdoor noise was investigated only for residents living in flats in each city. Kruskal–Wallis and Mann–Whitney U tests were used for comparisons between floors using Bonferroni’s correction for multiple comparisons. The residents living in the intermediate floor(s) showed higher annoyance ratings for road traffic and airplanes/helicopters in Seoul. In particular, the mean annoyance rating due to airplanes/helicopters in the intermediate floor(s) was statistically higher than those in the ground and top floors (*p* < 0.05 for both). In contrast, the residents in the intermediate floor(s) had lower annoyance ratings across the sources in London; however, there were no significant differences between floors (Kruskal–Wallis test *p* > 0.05). Similarly, the differences in annoyance ratings between floors were not significant in São Paulo (Kruskal–Wallis test *p* > 0.05) although the intermediate floor(s) tended to have slightly higher annoyance ratings than other floors. The mean annoyance ratings, %HA ratings, and the percentages of respondents hearing noise across the floors and cities are provided in the Supplementary Figures S1–S3.

### 3.2. Neighbour Noise Annoyance

**Figure 3:**
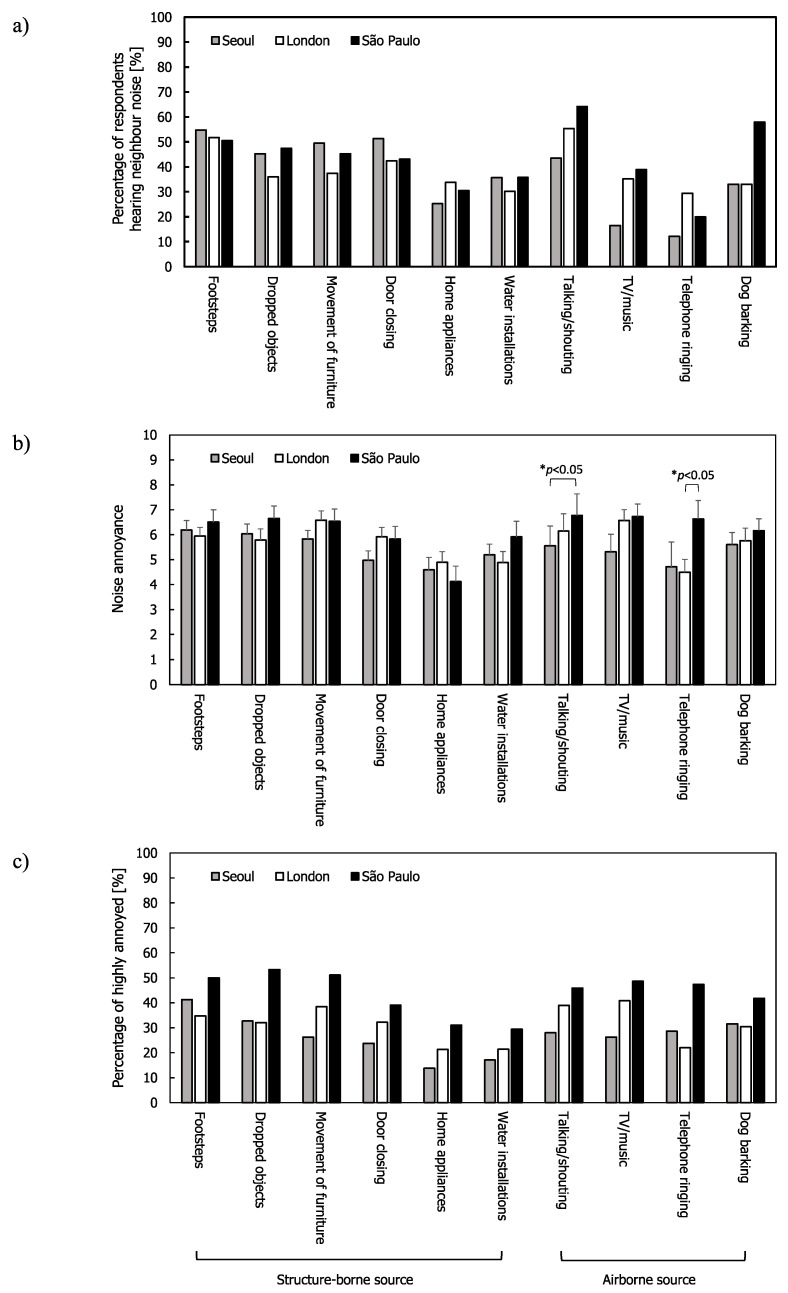
Neighbour noise: (**a**) Percentage of respondents hearing noise for all the respondents; (**b**) mean annoyance; (**c**) %HA ratings. Error bars indicate standard errors (* *p* < 0.05).

For neighbour noise, Figure 3 shows the mean annoyance and %HA ratings along with the percentage of respondents across the three cities. In detached houses, structure-borne noise sources were not heard and only a few respondents answered that they heard some airborne noise sources such as TV/music and dogs barking. Most noise sources in São Paulo had higher mean annoyance ratings and %HA ratings than in Seoul and London. In Seoul, the average percentage of the respondents hearing structure-borne sources (43.6%) was higher than for those hearing airborne sources (26.3%), whereas in São Paulo more people heard airborne noise sources (45.3%) than structure-borne noise sources (42.1%). In London, the average percentage of respondents hearing structure-borne and airborne sources were almost the same (38.6% and 38.3%, respectively). These differences between the cities indicate the importance of assessing dominant sources to inform the building design strategy to reduce annoyance. The participants were also asked whether they heard light switches as a structure-borne source in the surveys. However, this was not included in the analysis because very few respondents heard the noise (2, 11, and 1 in Seoul, London, and São Paolo, respectively).

In Seoul, footsteps were the most frequently heard noise source, followed by doors closing and the movement of furniture. Footsteps were the most annoying noise source in terms of mean annoyance and %HA ratings, while the movement of furniture, talking/shouting, and TV/music were the most annoying sources in London. This finding is in agreement with previous studies that reported footsteps as the most frequently heard noise source in Korean apartment buildings [44,53]. Carpets are not common in Korea and residents walk barefoot rather than wearing shoes; this leads to high maximum Fast time-weighted sound pressure levels at low-frequencies [54].

In London, the most frequently heard noises were footsteps and talking/shouting. Earlier studies in the UK reported that music, television, radio, and footfalls on the floor were the most commonly heard noises [43], and that footsteps, music, television, and radio were most frequently heard from above with music, television, radio, and voices being heard the most from the side [46]. One possible reason that television and radio are no longer as commonly heard could be due to increased listening on headphones and the decrease in real-time television viewing, such that viewing times in adjacent habitable rooms are no longer synchronised [55].

In São Paulo, talking/shouting and dogs barking were most frequently heard. Talking/shouting and telephone ringing were significantly higher than those in Seoul and London, respectively. São Paulo has ≈2.5 million owned dogs (although there are also many stray dogs [56]), giving a minimum dog:inhabitant ratio of ≈4.4 [56]. This is significantly higher than in London where there were ≈310,000 owned dogs in 2016 [57]. Although recent figures are not available for Seoul, it is assumed that there are fewer dogs per inhabitant than in London and São Paulo.

One-way ANOVA indicated that the only mean annoyance ratings that were different across the cities were for talking/shouting and telephone ringing ((F(2,190) = 2.907, *p* < 0.05 for talking/shouting and F(2,174) = 3.302, *p* < 0.05 for telephone ringing)). Post hoc comparisons (Tukey’s test) showed that the mean annoyance ratings for talking/shouting in Seoul and São Paulo were statistically different (*p* < 0.05) and that the mean annoyance rating caused by the telephone ringing in São Paulo was significantly greater than that in London (*p* < 0.05). This might indicate that the mid- to high-frequency airborne sound insulation (particularly against sources such as talking/shouting and telephones ringing) is lower than in the other two cities.

**Figure 4:**
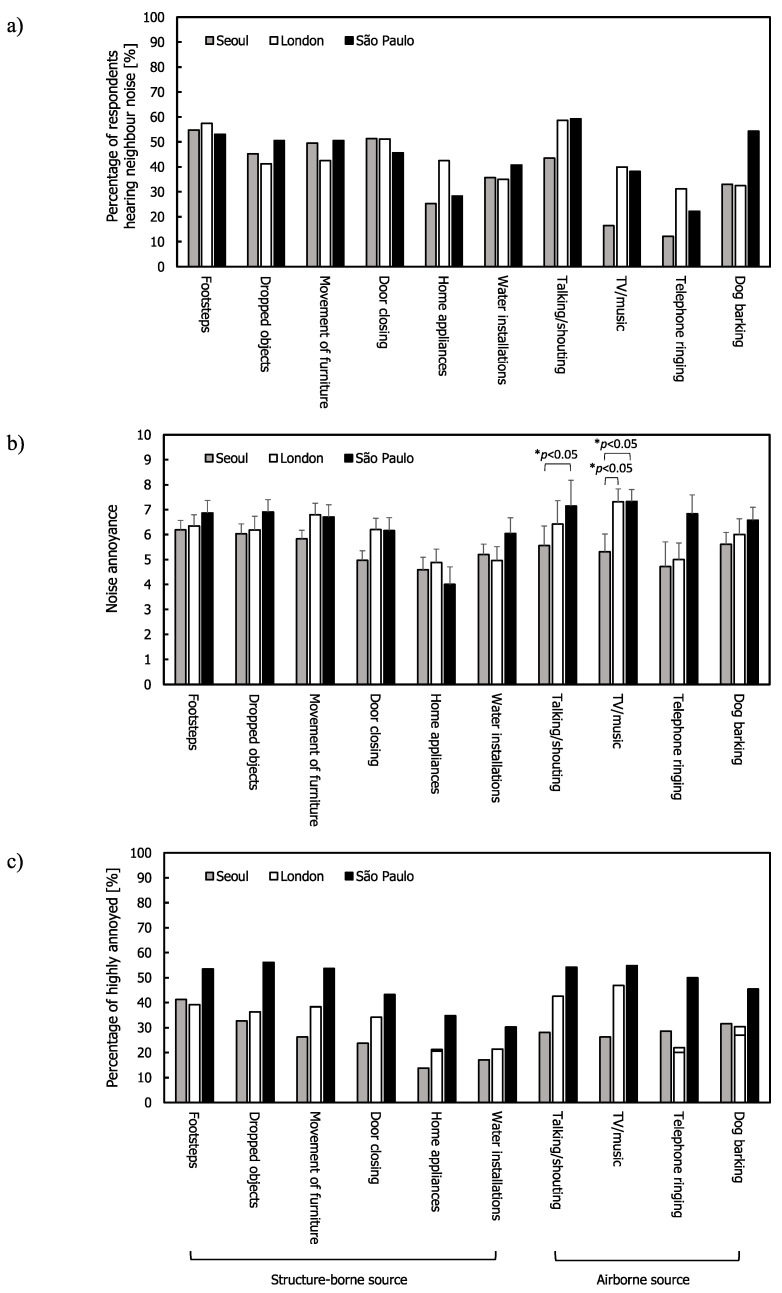
Neighbour noise: (**a**) Percentage of respondents hearing noise for the respondents living in flats; (**b**) mean annoyance; (**c**) %HA ratings. Error bars indicate standard errors (* *p* < 0.05).

Similar tendencies were found from residents living only in flats (Figure 4). The mean annoyance ratings and %HA ratings in São Paulo were higher than those in Seoul and London for most noise sources. Contrary to the results from all the respondents in Figure 3c, the residents in the three cities were more exposed to structure-borne sources (43.6%, 45.0%, and 44.9% for Seoul, London, and São Paulo, respectively) than airborne noise sources (26.3%, 40.6%, and 43.5% for Seoul, London, and São Paulo, respectively). Footsteps were the most frequently heard noise in Seoul followed by door closing, while footsteps and talking/shouting were most frequently heard in London and São Paulo. One-way ANOVA and the Kruskal–Wallis test (only for telephone ringing) indicated that the annoyance ratings of talking/shouting and TV/music were different across the three cities ((F(2,142) = 3.753, *p* < 0.05 for talking/shouting and F(2,79) = 4.233, *p* < 0.05 for TV/music). Post hoc comparisons (Tukey’s test) also showed that Seoul had lower mean annoyance ratings for talking/shouting than São Paulo (*p* < 0.05) and for TV/music than London and São Paulo (*p* < 0.05 for both).

**Figure 5:**
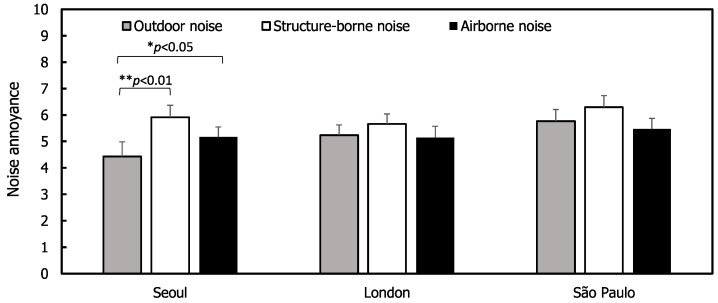
Comparisons of outdoor noise and indoor (structure-borne and airborne) noise. Error bars indicate standard errors (* *p* < 0.05, ** *p* < 0.01).

Paired samples *t*-tests were conducted to examine the differences between the annoyance ratings for indoor and outdoor sources. To make this comparison, the outdoor noise annoyance ratings were averaged for the four transportation sources, and the indoor noise ratings were averaged separately for structure-borne and airborne sources. Figure 5 shows that indoor sources were more annoying than outdoor sources in Seoul (*t*(38) = −4.10, *p* < 0.01 for structure-borne and *t*(42) = −2.37, *p* < 0.05 for airborne sources). However, the differences between annoyance ratings for indoor and outdoor sources were not statistically significant in London and São Paulo. This is consistent with the results of a UK survey in 2012, which reported that noise annoyance from road traffic and neighbours were similar [12]. However, an earlier UK survey from 1977 showed that when neighbour noise was heard, it resulted in greater annoyance than outdoor transportation noise [9]. This may indicate that changes have occurred in attitude to indoor and outdoor sources of noise over time.

### 3.3. Communal Noise Annoyance

**Figure 6:**
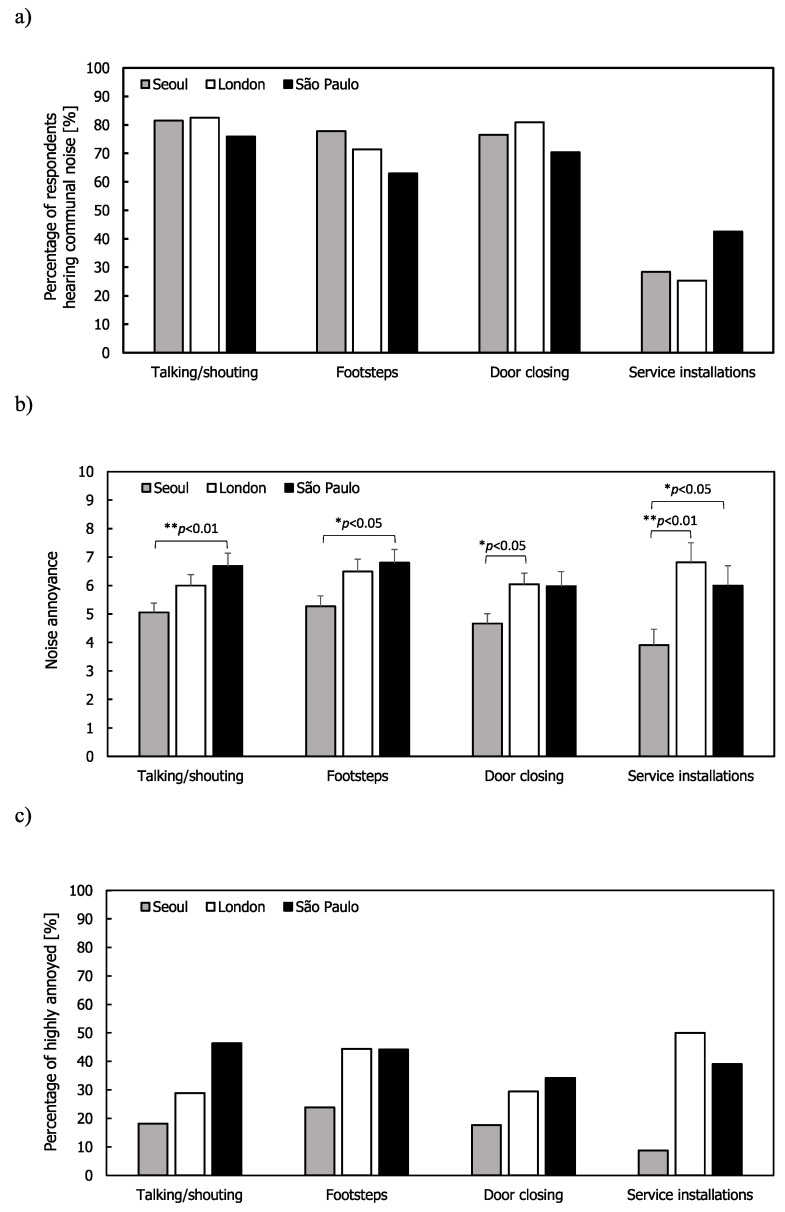
Communal noise: (**a**) Percentage of respondents hearing noise from communal area; (**b**) mean annoyance; (**c**) %HA ratings. Error bars indicate standard errors (* *p* < 0.05, ** *p* < 0.01).

The results for noise from communal areas in flats is shown in Figure 6. In general, the percentages of respondents were larger than 60% except for service installations and the mean annoyance ratings were similar across the sources for all the cities. In Seoul and São Paulo, talking/shouting and footsteps had higher mean annoyance ratings than doors closing and service installations. The mean annoyance and %HA ratings caused by the service installations were highest in London but the percentage of respondents hearing these sources was much lower than with the other sources. ANOVA was also used to investigate the annoyance ratings across the cities. It was found that the annoyance ratings were significantly different for all the sources ((F(2,157) = 4.665, *p* < 0.05 for talking/shouting; (F(2,142) = 4.404, *p* < 0.05 for footsteps; F(2,151) = 4.148, *p* < 0.05 for door closing; (F(2,59) = 5.260, *p* < 0.01 for service installation). The mean annoyance ratings for talking/shouting, footsteps, doors closing, and service installations in Seoul were significantly lower than those in London and São Paulo.

### 3.4. Cause of Noise, Relationship with Neighbours and Noise Complaints

Table 2 gives the responses to the question “Do you know which of your neighbours cause the noise?” In all three cities, respondents said that they could identify some of the neighbours causing the noise, the highest percentage being from Seoul (82.1%). In London and São Paulo, approximately 21% and 26%, respectively, said that they could identify all neighbours that were the cause of the noise.

**Figure 7:**
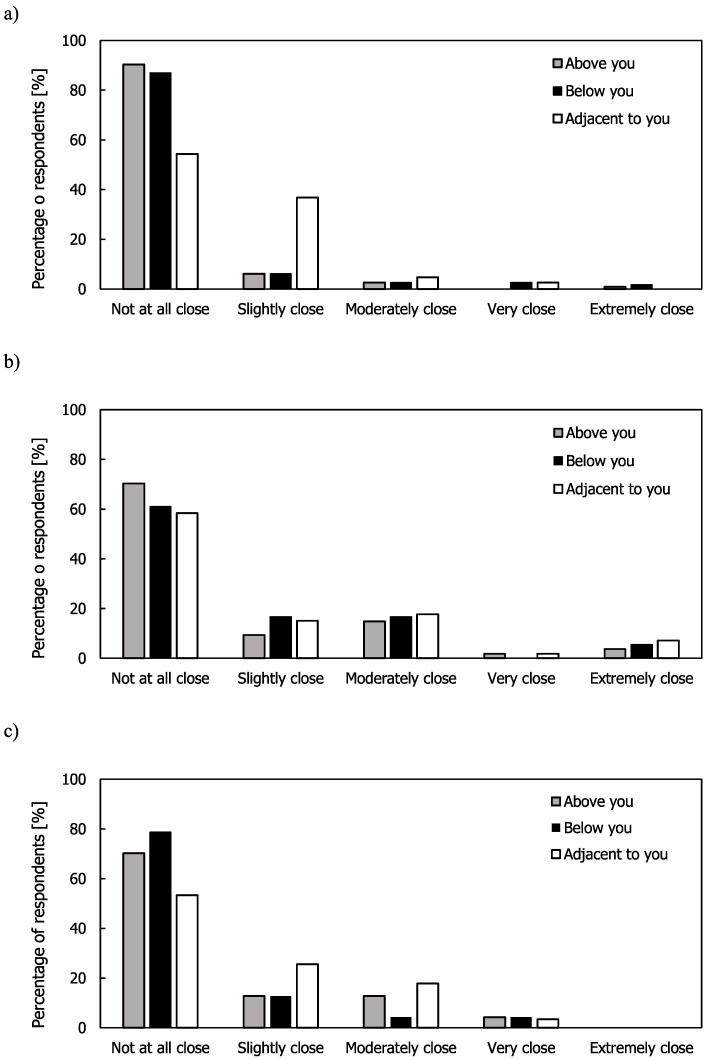
Relationships with neighbours in (**a**) Seoul; (**b**) London; and (**c**) São Paulo.

Respondents living in flats were asked to describe their relationship with neighbours that they identified as being the cause of the noise for neighbours that were above, below, and adjacent to them. Combining the results from all three cities, the majority responded that they were not at all close with these neighbours (see Figure 7).

In Seoul, almost all respondents lived in flats and had the highest percentage choosing “not at all close” (90.6%, 87.2%, and 55.1% for above you, below you, and adjacent to you, respectively). For adjacent neighbours, a larger percentage of respondents in Seoul (37.6%) indicated that they were “slightly close” compared to London (18.7%) and São Paulo (28.0%). It is not known whether this is a cultural difference or the fact that the majority of respondents were in flats that in Korea usually have similar floor plans with two units facing each other (the building types and building plans are likely to be more varied in London and São Paulo). Respondents in London and São Paulo had relationships that were closer than “slightly close” with the neighbours adjacent to them than the neighbours above or below them.

**Figure 8:**
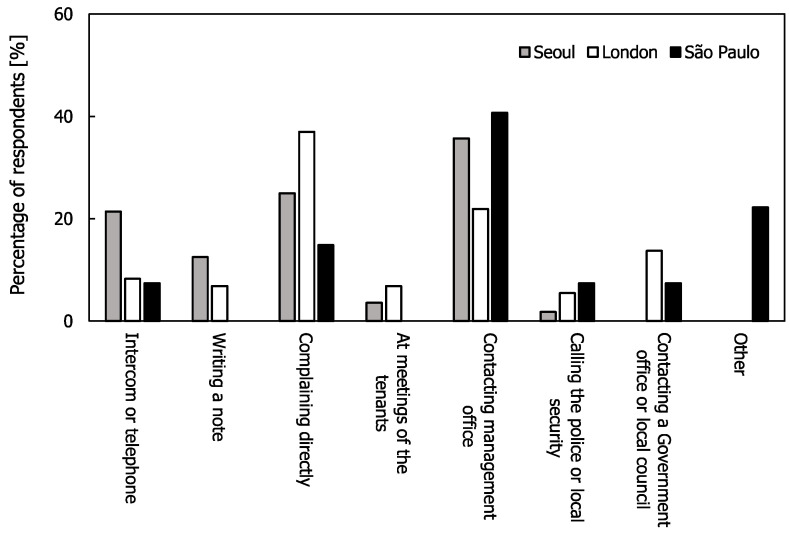
Methods used to complain to neighbours that were identified as being the cause of the noise.

In Seoul, London, and São Paulo, 47.9%, 36.6%, and 52.1% of the respondents, respectively, complained to their neighbours about their noise. Figure 8 shows the methods that were used to complain to these neighbours. In Seoul and São Paulo, the most common approach was to contact the management office, whereas in London it was to complain directly to the neighbours. In São Paulo, the percentage who made a direct complaint to the neighbours was the lowest of all three cities; in addition, 20% answered that they complained in other ways, such as contacting security guards, charities, or leaving messages for neighbours.

### 3.5. Effects of Non-Acoustic Factors on Noise Annoyance and Coping Strategies

The effect on noise annoyance and coping strategies due to the following non-acoustic factors were assessed: noise sensitivity, level of education, income, and the group of people living in the same dwelling. Firstly, the respondents were divided into two groups with low and high noise-sensitivity scores to examine the effect of noise sensitivity on noise annoyance and coping strategies. The classifications of respondents were carried out using the median values as a cut-off point; hence, the respondents whose noise-sensitivity scores are above the median were classified as the high noise-sensitivity group. Secondly, the respondents were considered in two groups according to their level of education (school level or university level). Thirdly, the respondents were categorised into two groups according to their annual house income (above £53,500 or below £53,500). Lastly, two sub-groups of respondents were considered: those living with children, and those not living with children.

**Figure 9:**
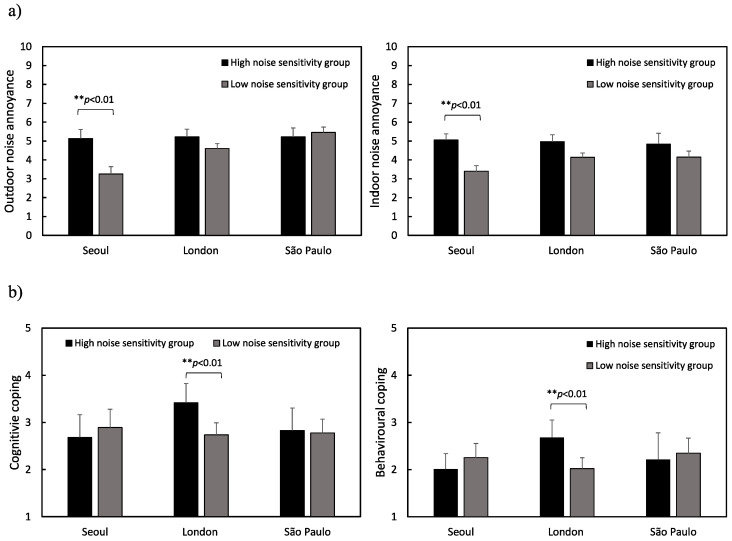
The mean outdoor and neighbour noise annoyance ratings (**a**) and cognitive and behavioural coping strategy ratings (**b**) across the low and high noise-sensitivity groups with error bars indicating standard errors (** *p* < 0.01).

Figure 9 shows the average outdoor and indoor noise annoyance ratings as well as the cognitive and behavioural coping strategy ratings across the low and high noise-sensitivity groups for the three cities. The high noise-sensitivity groups showed higher indoor and outdoor noise annoyance ratings in all three cities except for outdoor noise annoyance in São Paulo. Independent *t*-tests show that the high noise-sensitivity group in Seoul had significantly higher indoor and outdoor noise annoyance ratings than the low noise-sensitivity group (*t*(50) = 3.033, *p* < 0.01 for outdoor noise and *t*(76) = 3.701, *p* < 0.01 for indoor noise). This finding is in agreement with previous studies that found there to be a significant effect of noise sensitivity on annoyance and emotional ratings [24,25,26,58,59]. However, in London and São Paulo, the differences between the two groups were not significant.

In terms of coping strategy ratings, the high noise-sensitivity group in London more frequently used cognitive and behavioural coping strategies compared to those in the low noise-sensitivity group. In London, the differences in coping strategy scores between the low and high noise-sensitivity groups was significant (*t*(111) = 4.489, *p* < 0.01 for cognitive coping and *t*(111) = 4.533, *p* < 0.01 for behavioural coping). The differences between the low and high noise-sensitivity groups in Seoul and São Paulo were small and not statistically significant.

Most non-acoustic factors, such as education, house income, and gender, had a negligible effect on noise annoyance and coping strategy ratings. Differences in noise annoyance and coping strategy ratings were not significant across education, house income, and gender groups in all the cities. However, compared to São Paulo, people living with children in Seoul and London had lower indoor and outdoor noise annoyance ratings, and lower cognitive and behavioural coping strategy ratings. The difference in cognitive coping strategy scores between the groups was significant in Seoul (*t*(86) = 1.897, *p* < 0.05). This supports the finding from a previous study in Korea [60], which suggested that residents with children were more likely to be empathetic to children’s noise from upstairs. A recent study on floor impact noise showed that people living with one or more children showed the highest empathy ratings [59].

### 3.6. Relationships between Noise Annoyance and Coping Strategy

People that are exposed to noise tend to develop cognitive and behavioural coping strategies [7,61,62]. Correlation analysis was used to examine the relationships between noise annoyance and coping strategy. Before the calculations, the outdoor noise annoyance ratings were averaged for four noise sources and the indoor noise annoyance ratings were also averaged for ten neighbour noise sources. The resulting correlation coefficients are listed in Table 3. All the correlation coefficients were positive, indicating that an increase in the noise annoyance rating leads to more frequent use of coping strategies. This is in agreement with previous findings on the positive relationships between noise annoyance and coping capacity [24,63,64]. Moreover, annoyance with neighbour noise showed stronger relationships with coping strategy ratings compared to annoyance with outdoor noise. This might be because neighbour noise sources are less predictable as to when they will occur. Furthermore, when compared to sources such as steady road traffic noise, they are intermittent, such that residents may develop more coping strategies. Previous studies [65,66] reported that intermittent stress exposure stimulates the development of stress coping skills. In Seoul, the indoor noise annoyance rating was significantly correlated with both the cognitive and behavioural coping strategy ratings, while the outdoor noise annoyance rating was correlated only with the behavioural coping strategy rating. In London, both the indoor and outdoor annoyance ratings were significantly correlated with both the cognitive and behavioural coping strategy ratings. However, in São Paulo, only the indoor noise annoyance rating was significantly correlated with the coping strategies.

### 3.7. Moderation Effects of Noise Sensitivity

**Figure 10:**
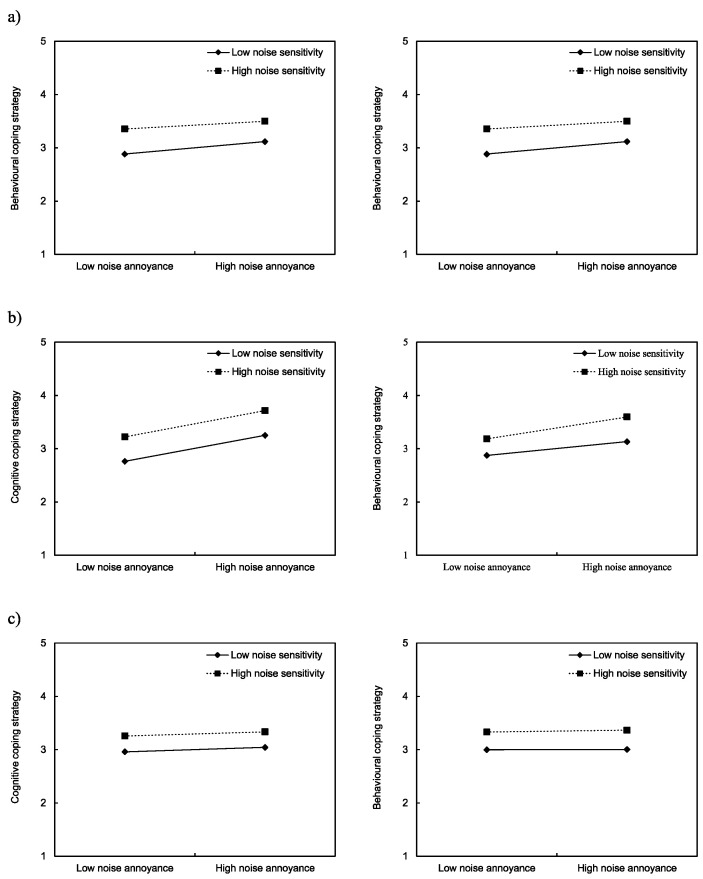
Interaction between neighbour noise annoyance and noise sensitivity on cognitive and behavioural coping in (**a**) Seoul; (**b**) London; and (**c**) São Paulo.

Previous studies [8,67,68,69] reported that noise sensitivity moderates the effect of noise on annoyance. In addition, several studies have reported causal relationships between noise annoyance and coping strategies [24,61,63]. However, it was not clear whether noise sensitivity moderates the relationship between noise annoyance and coping strategies. Thus, the present study investigated the moderating effect of noise sensitivity on the relationship between noise annoyance and coping strategy. It was assumed that noise sensitivity might alter the causal relationship between noise annoyance and coping strategies. In order to examine the moderation effect of noise sensitivity, a two-way interaction technique was employed [49]. Figure 10 shows that respondents with a high noise sensitivity developed more coping strategies, both at low and high noise annoyance, in all three cities. It was observed that the effects of noise annoyance on coping strategy were similar for the low and high noise-sensitivity groups. However, the regression analysis indicates that the interaction term was not significant in any of the three cities by confirming the insignificant moderation effects of noise sensitivity on relationships between noise annoyance and coping strategies.

### 3.8. Relationship between Closeness with Neighbours and Coping Strategy

Attitudinal variables, such as attitude towards noise source or authorities, have been reported to affect outdoor noise annoyance and coping capacity [7,61,63]. Recently, closeness with neighbours was also suggested as an attitudinal variable affecting coping strategies to indoor noise [24,62,70]. Hence, in the present study, it was hypothesised that closeness with neighbours might affect coping strategies. Correlation coefficients between closeness with neighbours and coping strategies are given in Table 4. The results show that the influence of closeness with neighbours on coping strategy was limited. In São Paulo, closeness with neighbours was negatively correlated with cognitive coping strategies (*r*(68) = −0.236, *p* < 0.05), implying that a better relationship with neighbours reduced the development of cognitive coping strategies. In contrast, the relationships between closeness with neighbours and coping strategy were not significant in Seoul and London. This can be explained by limited variations of the closeness with neighbours’ ratings, plotted in Figure 4. In the present study, only one direct question was used; however, it was not sufficient to successfully measure the closeness with neighbours. Therefore, multiple questions that were used in another study [24] could be applied in the future.

### 3.9. Comparison of Noise Annoyance for Occupants of Flats and Houses

**Figure 11:**
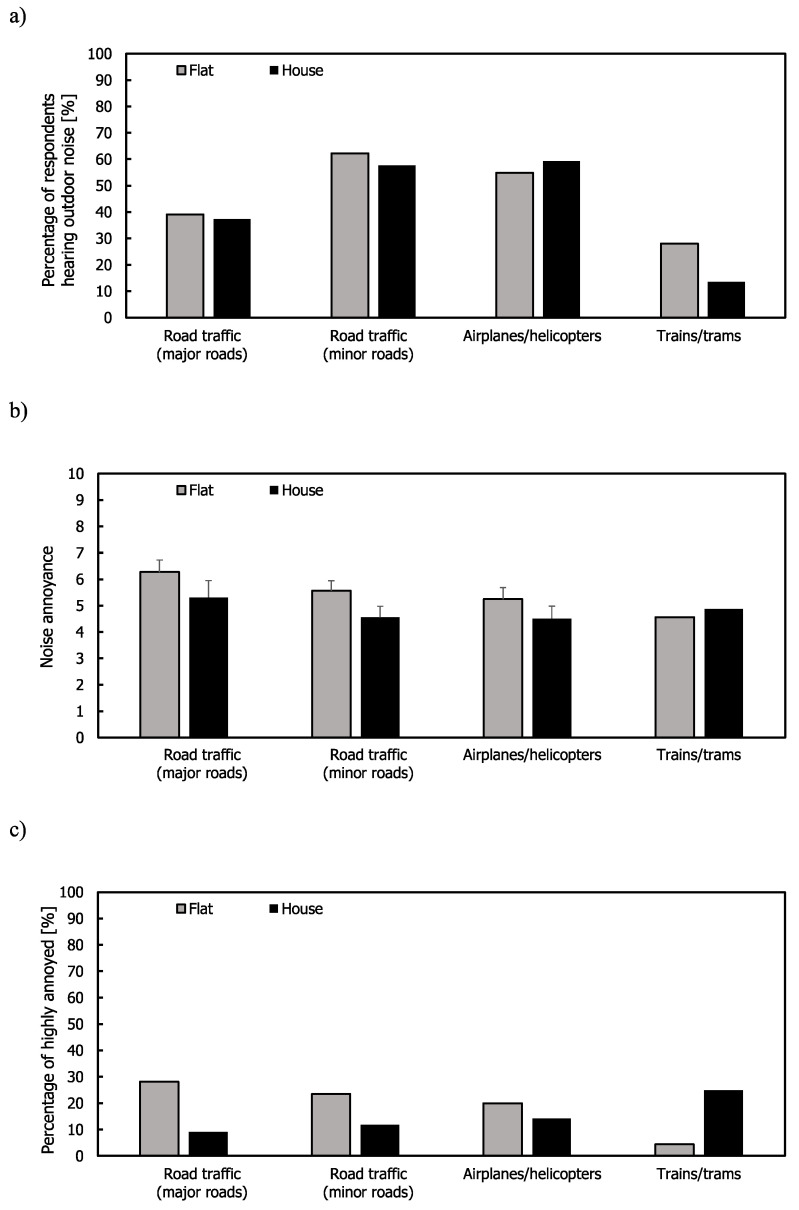
Outdoor noise for flats and houses in London: (**a**) Percentage of respondents hearing noise; (**b**) mean annoyance; (**c**) %HA ratings. Error bars indicate standard errors.

A comparison is now made of the outdoor and indoor noise annoyance ratings in flats and houses (defined as semi-detached and terraced houses). The number of respondents living in houses in Seoul and São Paulo were small, so this comparison was only performed using the data from London. Figure 11 shows that residents in flats had greater mean annoyance and %HA ratings than those living in houses, except for noise from trains/trams. However, the differences between flats and houses were not statistically significant. Flats and houses showed similar percentages of respondents hearing outdoor noise and more than half of the respondents in flats and houses heard road traffic noise from minor roads and noise from airplanes/helicopters.

**Figure 12:**
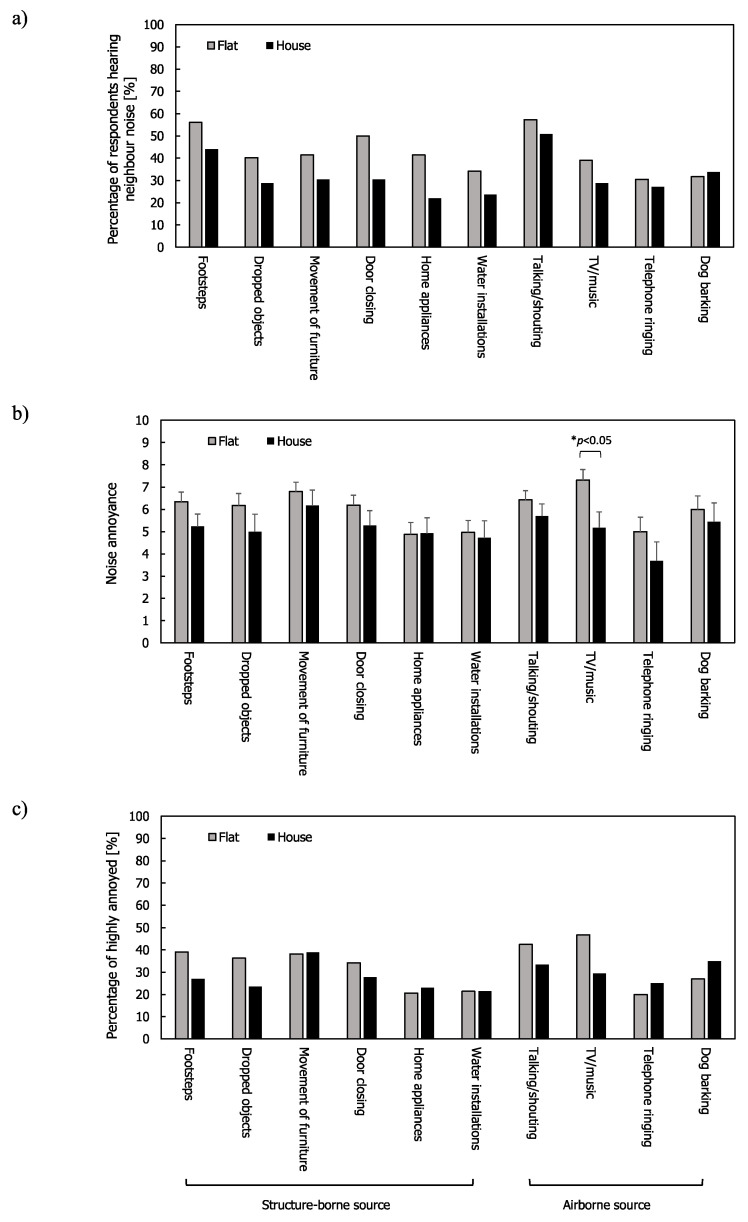
Neighbour noise for flats and houses in London: (**a**) Percentage of respondents hearing noise; (**b**) mean annoyance; (**c**) %HA ratings. Error bars indicate standard errors (* *p* < 0.05).

As shown in Figure 12, the respondents from flats in London tended to have greater noise annoyance ratings compared to those in houses. This is in line with a previous study from the UK, which reported that residents in flats were more bothered by neighbour noise than those living in houses [9]. In addition, the percentages of respondents hearing neighbour noise in flats were larger than those in houses. However, in the present study, the difference was only statistically significant in the annoyance rating due to TV/music (F(1,47) = 50.654, *p* < 0.05). The insignificant differences for other noise sources might be due to the small sample size; this could be investigated further with more samples in future studies.

### 3.10. Limitations

As with all studies, there are some limitations to consider. First, in contrast to previous studies on outdoor noise annoyance [3,4,14], it was not possible to link annoyance with the measured noise levels. Most indoor noise sources are intermittent, and it is rarely possible to constantly monitor sound pressure levels in several rooms occupied by the respondents. For this reason, the study only concerns subjective responses and other non-acoustic factors without objective noise measurements. Recent studies have suggested the use of smartphone microphones for noise measurements [71,72,73] and noise mapping [74] and this could be considered in future indoor noise studies to identify relationships between objective measures and subjective responses. Ideally, such data would be combined with information on the sound insulation of the façade, separating walls and floors; this could help to identify suitable levels of sound insulation for building regulations. Several limitations were related to sampling of the participants. The samples were skewed towards residents with a high educational background in Seoul and São Paulo due to the snowball sampling method. More than 90% of the respondents in the two cities had at least a university degree, whereas only 63% of the respondents in London had a university degree. The sample was also not a representative sample of the population, with different dwelling types. Specifically, the proportions of respondents living in flats were around 98%, 59.0%, and 75%, respectively, in Seoul, London, and São Paulo, whereas flats represent approximately 63%, 50%, and 28% in each city. Additionally, only participants who heard the noise were asked to rate their noise annoyance and this resulted in small sample sizes across the noise sources. Thus, larger and more diverse samples would be helpful in future studies. Lastly, information on buildings, such as floor and wall structures, building year, and the floor the respondents live on, were not available in the present study. Future research might find further insights if more information was available on the building construction.

## 4. Conclusions

This study investigated the annoyance of indoor and outdoor noise people feel inside their homes using questionnaire surveys from respondents in Seoul, London, and São Paulo. Neighbour noise was found to be more annoying than transportation noise in Seoul but equally annoying in London and São Paulo. The average percentage of respondents hearing structure-borne sources compared to those hearing airborne sources differed between the three cities: in Seoul, structure-borne sources were more commonly heard; in São Paulo, it was airborne sources; and in London, both were heard similarly. In Seoul, footsteps were the most frequently heard source of neighbour noise, and were the most annoying source, whereas the movement of furniture, talking/shouting, and TV/music were the most annoying sources in London. Most neighbour noise sources in São Paulo gave rise to higher annoyance ratings than Seoul and London. Respondents said that they could identify some of the neighbours causing the noise, particularly in Seoul where noise sensitivity was also found to significantly affect neighbour noise annoyance. The effect of living with or without children, education level, and annual house income in all three cities had little effect on noise annoyance and coping strategies.

Indoor noise annoyance was found to have stronger relationships with cognitive and behavioural coping strategies than outdoor noise annoyance. Coping strategies were not affected by relationships with neighbours; however, more detailed questions are necessary to assess residents’ attitude towards their neighbours. The findings indicate that neighbour noise in residential buildings is a significant source of noise annoyance. Further work to understand the effects of neighbour noise has the potential to improve regulatory practice on sound insulation.

## Figures and Tables

**Table 1:** Information about the participants from each site (last column indicates the results of the chi-square (χ^2^) test; ** *p* < 0.01).

Personal Characteristics	Seoul	London	São Paulo	Total	χ^2^ Test
Gender					*p* > 0.05
Male	58 (49.6%)	72 (50.0%)	45 (44.1%)	175 (48.2%)	
Female	59 (50.4%)	72 (50.0%)	57 (55.9%)	188 (51.8%)	
Total	117	144	102	363	
Age (years)					*p* > 0.05
18 to 35	57 (48.7%)	49 (34.0%)	50 (49.0%)	156 (43.0%)	
36 to 50	43 (36.8%)	50 (34.7%)	25 (24.5%)	118 (32.5%)	
51 to 64	16 (13.7%)	41 (28.5%)	23 (22.5%)	80 (22.0%)	
65 or over	1 (0.9%)	4 (2.8%)	4 (3.9%)	9 (2.5%)	
Type of dwelling					**
Flats	115 (98.3%)	85 (59.0%)	76 (74.54%)	276 (76.0%)	
Semi-detached house	1 (0.9%)	28 (19.4%)	17 (16.7%)	46 (12.7%)	
Terraced house	0 (0.0%)	26 (18.1%)	2 (2.0%)	28 (7.7%)	
Detached house	1 (0.9%)	5 (3.5%)	7 (6.9%)	13 (3.6%)	
Room where most time is spent during the day					*p* > 0.05
Living room	77 (65.8%)	100 (69.4%)	70 (68.6%)	247 (68.0%)	
Home office	13 (11.1%)	8 (5.6%)	12 (11.8%)	33 (9.1%)	
Bedroom	27 (23.1%)	36 (25.0%)	20 (19.6%)	83 (22.9%)	
Education					**
School level	14 (12.0%)	54 (37.5%)	4 (3.9%)	72 (19.8%)	
University level	103 (88.0%)	90 (62.5%)	98 (96.1%)	291 (80.2%)	
Employment					**
Full time	55 (47.0%)	64 (44.4%)	65 (63.7%)	184 (50.7%)	
Part time	15 (12.8%)	20 (13.9%)	7 (6.9%)	42 (11.6%)	
Self-employed	5 (4.3%)	16 (11.1%)	20 (19.6%)	41 (11.3%)	
Unemployed	4 (3.4%)	14 (9.7%)	2 (2.0%)	20 (5.5%)	
Student	12 (10.3%)	12 (8.3%)	7 (6.9%)	31 (8.5%)	
Homemaker	26 (22.2%)	10 (6.9%)	0 (0.0%)	36 (9.9%)	
Retired	0 (0.0%)	8 (5.6%)	1 (1.0%)	9 (2.5%)	
Annual household income					**
<£10,700 (₩ 30,000,000, R$10,500)	15 (12.8%)	12 (8.3%)	16 (15.7%)	43 (11.8%)	
£10,700–£53,500 (₩ 80,000,000, R$52,500)	68 (58.1%)	77 (53.5%)	33 (32.4%)	178 (49.0%)	
£53,500–£107,700 (₩ 100,000,000, R$105,000)	26 (22.2%)	33 (22.9%)	21 (20.6%)	80 (22.0%)	
>	3 (2.6%)	5 (3.5%)	27 (26.5%)	35 (9.6%)	
Not known	5 (4.3%)	17 (11.8%)	5 (4.9%)	27 (7.4%)	
People you live with					**
No one	16 (13.7%)	36 (25.0%)	16 (15.7%)	68 (18.7%)	
With people who are not your family	2 (1.7%)	25 (17.4%)	9 (8.8%)	36 (9.9%)	
With family including children under 12 years old	31 (26.5%)	29 (20.1%)	12 (11.8%)	72 (19.8%)	
With family including children 12 years old	17 (14.5%)	24 (16.7%)	12 (11.8%)	53 (14.6%)	
With family but without children	51 (43.6%)	30 (20.8%)	53 (52.0%)	134 (36.9%)	

**Table 2:** Identification of neighbours that cause noise (%).

	None	Some of Them	All of Them	I don’t Have Noisy Neighbours
Seoul	0.9	82.1	2.6	14.5
London	4.5	56.3	20.5	18.8
São Paulo	16.7	47.8	25.6	10.0

**Table 3:** Correlation coefficients between noise annoyance and coping capacity (** *p* < 0.01).

Coping Strategy	Seoul		London		São Paulo	
	Outdoor	Indoor	Outdoor	Indoor	Outdoor	Indoor
Cognitive	0.179	0.408 **	0.528 **	0.496 **	0.170	0.389 **
Behavioural	0.385 **	0.456 **	0.359 **	0.540 **	0.206	0.361 **

**Table 4:** Correlation coefficients between closeness with neighbours and coping capacity (* *p* < 0.01).

	Seoul	London	São Paulo
Cognitive coping	−0.164	−0.046	−0.236 *
Behavioural coping	0.085	0.003	−0.087

## Supplementary Materials

The following are available online at https://www.mdpi.com/1660-4601/17/16/6005/s1, Figure S1: Outdoor noise in Seoul across the floor on which the respondents live (a) percentage of respondents hearing noise, (b) mean annoyance ratings, (c) %HA ratings. Error bars indicate standard errors (* *p* < 0.05), Figure S2: Outdoor noise in London across the floor on which the respondents live (a) percentage of respondents hearing noise, (b) mean annoyance ratings, (c) %HA ratings. Error bars indicate standard errors, Figure S3: Outdoor noise in São Paulo across the floor on which the respondents live (a) percentage of respondents hearing noise, (b) mean annoyance ratings, (c) %HA ratings. Error bars indicate standard errors, Text Material S1: Questionnaire.
