# Supplementary material for: Attitudes to Noise Inside Dwellings in Three Megacities: Seoul, London, and São Paulo
Source: Int J Environ Res Public Health. 2020 Aug 18;17(16):6005. doi: 10.3390/ijerph17166005 (PMC7460320; doi:10.3390/ijerph17166005)
Supplement: Supplementary file 1 [file ijerph-17-06005-s001.pdf]

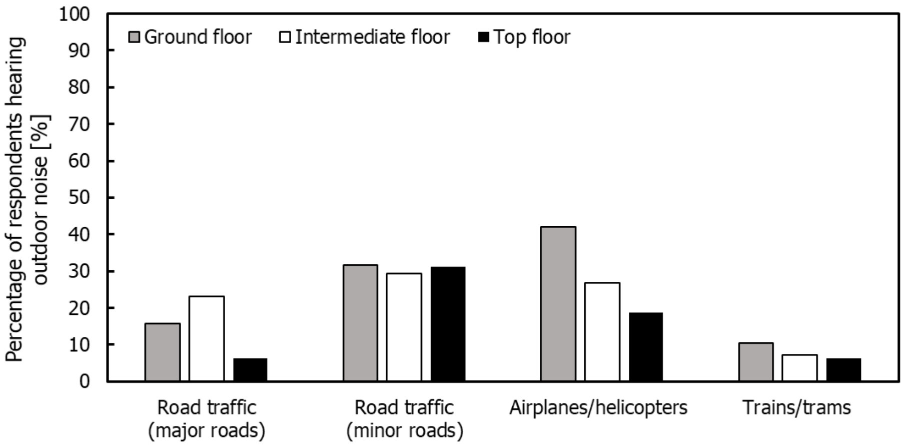

(a)

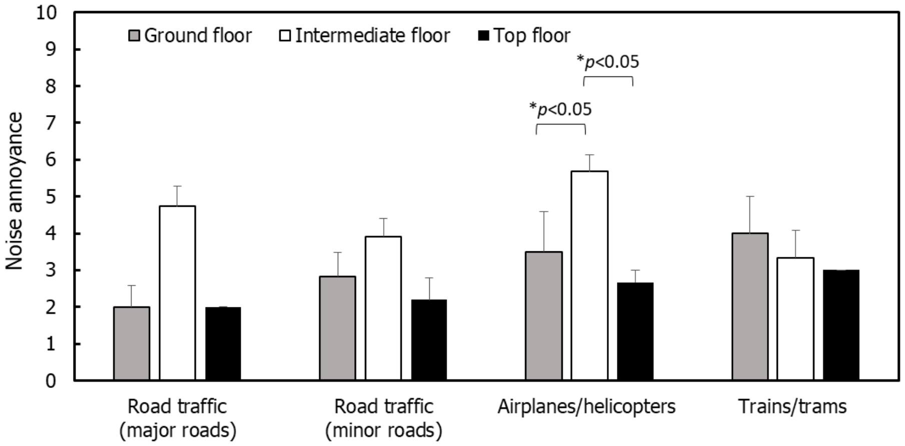

(b)

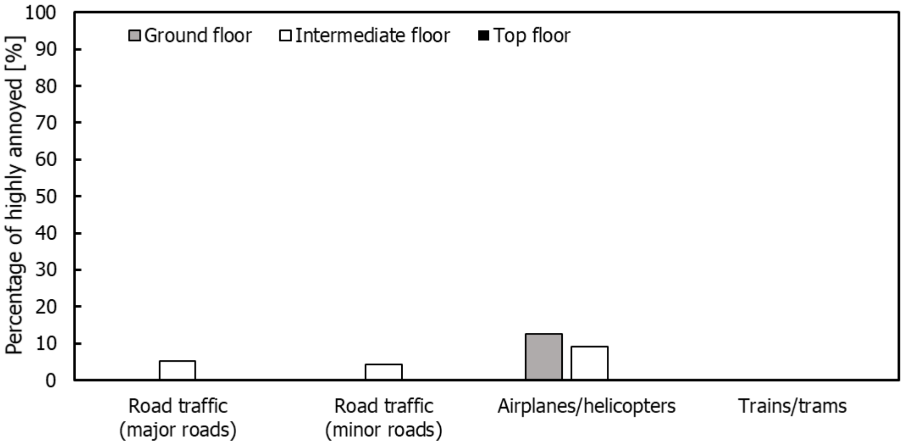

(c)

Figure S1. Outdoor noise in Seoul across the floor on which the respondents live (a) percentage of respondents hearing noise, (b) mean annoyance ratings, (c) %HA ratings. Error bars indicate standard errors (* $p < 0.05$).

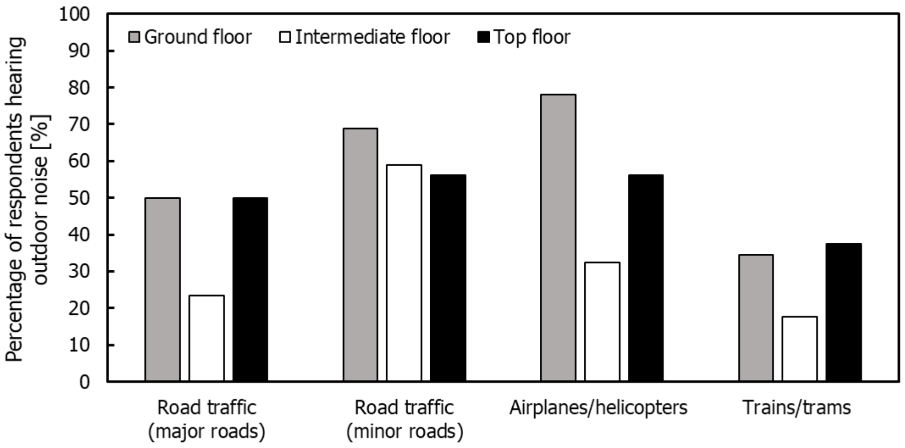

(a)

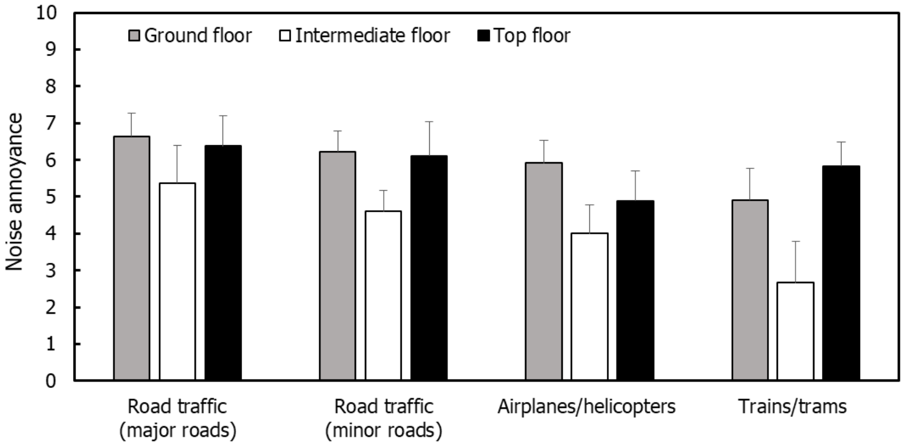

(b)

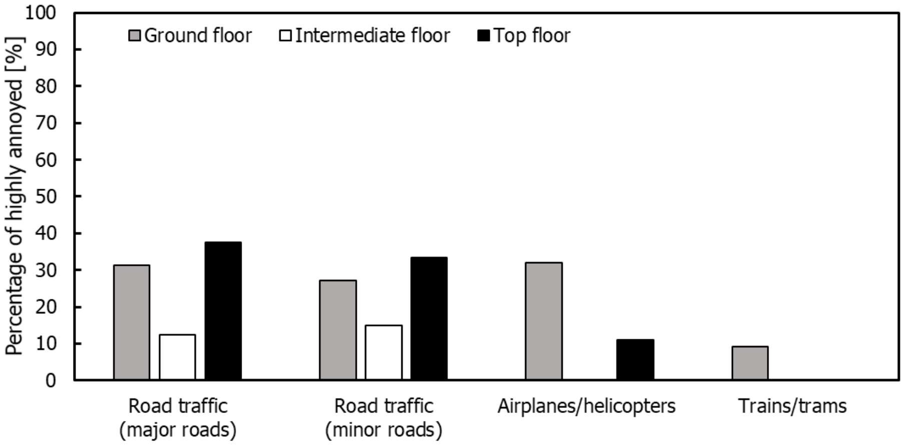

(c)

Figure S2: Outdoor noise in London across the floor on which the respondents live (a) percentage of respondents hearing noise, (b) mean annoyance ratings, (c) %HA ratings. Error bars indicate standard errors.

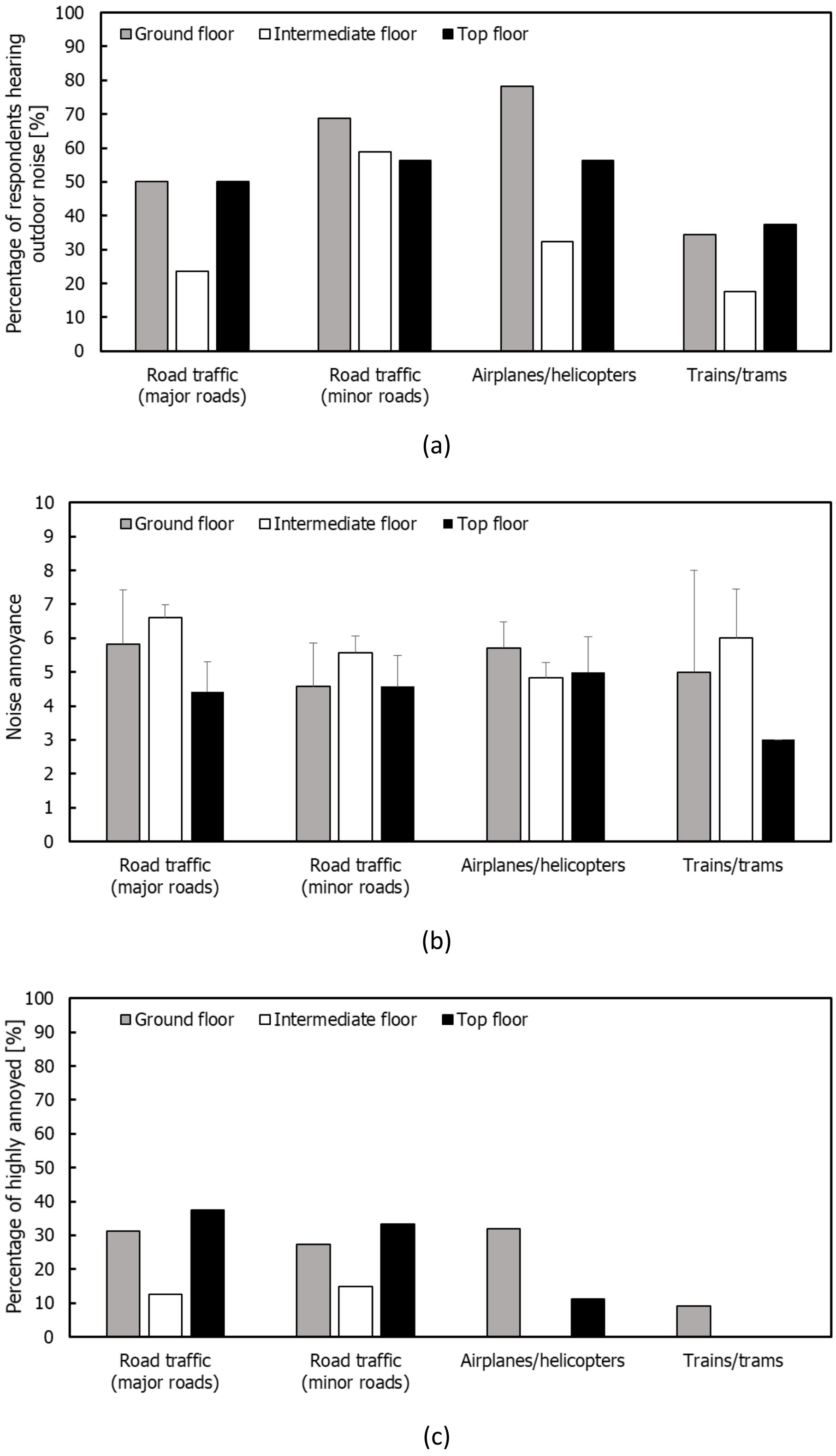

Figure S3: Outdoor noise in São Paulo across the floor on which the respondents live (a) percentage of respondents hearing noise, (b) mean annoyance ratings, (c) %HA ratings. Error bars indicate standard errors.

Text Material S1: Questionnaire.

INFORMATION ABOUT YOUR HOME

1. **How satisfied are you with your home in terms of:**
   a. **Location to amenities (e.g. shops, parks)**
   ( ) not at all ( ) slightly ( ) moderately ( ) very ( ) extremely
   b. **Transport links**
   ( ) not at all ( ) slightly ( ) moderately ( ) very ( ) extremely
   c. **Safety of the neighbourhood**
   ( ) not at all ( ) slightly ( ) moderately ( ) very ( ) extremely
   b. **Number of rooms**

   ( ) not at all ( ) slightly ( ) moderately ( ) very ( ) extremely
   c. **Size of the rooms**

   ( ) not at all ( ) slightly ( ) moderately ( ) very ( ) extremely

2. **What is your postcode? _____________** (Note: If you don't wish to tell us your full postcode, the first 3 or 4 letters/numbers are sufficient.)

3. **Please describe the type of dwelling**
   a. Flat/ studio
   b. Semi-detached house
   c. Terraced house
   d. Detached house

FOR FLATS/ STUDIOS ONLY:

3.a. On which floor do you live?
   a. Top floor
   b. Intermediate floor
   c. Ground floor

FOR FLATS/STUDIOS AND HOUSES:

4. **How long have you been living in this flat/studio/house?**
   a. Less than 1 year
   b. Between 1 and 5 years
   c. Over 5 years

IF OVER 5 YEARS, GO TO 4a

4a Please tell us how many years you have been living in this flat/studio/house.
You can give an approximate number of years.

_____

5. **In what part of your flat/ house do you spend most of your time during the day?**
   ( ) Living room ( ) Home office ( ) Bedroom

<u>WE ARE GOING TO ASK THESE QUESTIONS SEPARATELY FOR: FLAT/LIVING ROOM, FLAT/ HOME OFFICE, FLAT/ BEDROOM, HOUSE/ LIVING ROOM, HOUSE/ HOME OFFICE, HOUSE/ BEDROOM, DEPENDING ON THE ANSWERS THEY GAVE IN THE PREVIOUS QUESTIONS</u>

<u>NOISE FROM OUTSIDE YOUR HOME*</u>

6. **When you are in your bedroom/ living room/ home office with the windows closed, do you hear noise from outside your home (e.g. major and minor roads, airplanes, helicopters, trains, trams)?** Yes/No

<u>IF YES, GO TO 7. IF NO, GO TO 11</u>

7. **Thinking about when you are in your bedroom/ living room/ home office with the windows closed, do you hear road traffic noise from major roads (motorways, dual carriageways and A roads)?** Yes/No

<u>IF YES, GO TO 7a</u>

7.a How much does noise from major roads (motorways, dual carriageways and A roads) annoy you?

This uses a scale from 0-to-10. If you are not annoyed at all choose 0; if you are extremely annoyed choose 10; otherwise, choose a number between 0 and 10, that represents your annoyance.

0 = Not at all annoyed										10 = Extremely annoyed
**0**	**1**	**2**	**3**	**4**	**5**	**6**	**7**	**8**	**9**	**10**

8. **Thinking about when you are in your bedroom/ living room/ home office with the windows closed, do you hear road traffic noise from minor roads (B roads, minor roads, cul-de-sacs)?** Yes/No

<u>IF YES, GO TO 8a</u>

8.a How much does noise from minor roads (B roads, minor roads, cul-de-sacs) annoy you?

This uses a scale from 0-to-10. If you are not annoyed at all choose 0; if you are extremely annoyed choose 10; otherwise, choose a number between 0 and 10, that represents your annoyance.

0 = Not at all annoyed		10 = Extremely annoyed

**0**	**1**	**2**	**3**	**4**	**5**	**6**	**7**	**8**	**9**	**10**

9. **Thinking about when you are in your bedroom/ living room/ home office with the windows closed, do you hear noise from airplanes and/or helicopters?** Yes/No

IF YES, GO TO 9a

9.a How much does noise from airplanes and/or helicopters annoy you?

This uses a scale from 0-to-10. If you are not annoyed at all choose 0; if you are extremely annoyed choose 10; otherwise, choose a number between 0 and 10, that represents your annoyance.

0 = Not at all annoyed		10 = Extremely annoyed

**0**	**1**	**2**	**3**	**4**	**5**	**6**	**7**	**8**	**9**	**10**

10. **Thinking about when you are in your bedroom/ living room/ home office with the windows closed, do you hear noise from trains and/or trams?** Yes/No

IF YES, GO TO 10a

10.a How much does noise from trains and/or trams annoy you?

This uses a scale from 0-to-10. If you are not annoyed at all choose 0; if you are extremely annoyed choose 10; otherwise, choose a number between 0 and 10, that represents your annoyance.

0 = Not at all annoyed		10 = Extremely annoyed

**0**	**1**	**2**	**3**	**4**	**5**	**6**	**7**	**8**	**9**	**10**

NOISE FROM COMMUNAL AREAS IN YOUR BUILDING*(ONLY FOR FLATS)

11. **When you are in your bedroom/ living room/ home office with the windows closed, do you hear noise from communal areas in your building (e.g. from talking, shouting, footsteps, jumping, running, doors closing)?** Yes/No

IF YES, GO TO 12. IF NO, GO TO 18

12. **Thinking about when you are in your bedroom/ living room/ home office with the windows closed, do you hear noise from talking and/or shouting that come from communal areas in your building?** Yes/No

IF YES, GO TO 12a

12.a How much does noise from talking and/or shouting that come from communal areas in your building annoy you?

This uses a scale from 0-to-10. If you are not annoyed at all choose 0; if you are extremely annoyed choose 10; otherwise, choose a number between 0 and 10, that represents your annoyance.

0 = Not at all annoyed										10 = Extremely annoyed
**0**	**1**	**2**	**3**	**4**	**5**	**6**	**7**	**8**	**9**	**10**

13. **Thinking about when you are in your bedroom/ living room/ home office with the windows closed, do you hear noise from footsteps and/or jumping and/or running that come from communal areas in your building?** Yes/No

IF YES, GO TO 13a

13.a How much does noise from footsteps and/or jumping and/or running that come from communal areas in your building annoy you?

This uses a scale from 0-to-10. If you are not annoyed at all choose 0; if you are extremely annoyed choose 10; otherwise, choose a number between 0 and 10, that represents your annoyance.

0 = Not at all annoyed		10 = Extremely annoyed

0	1	2	3	4	5	6	7	8	9	10

14. **Thinking about when you are in your bedroom/ living room/ home office with the windows closed, do you hear noise from doors closing that come from communal areas in your building?** Yes/No

IF YES, GO TO 14a

14.a How much does noise from doors closing that come from communal areas in your building annoy you?

This uses a scale from 0-to-10. If you are not annoyed at all choose 0; if you are extremely annoyed choose 10; otherwise, choose a number between 0 and 10, that represents your annoyance.

0 = Not at all annoyed										10 = Extremely annoyed
**0**	**1**	**2**	**3**	**4**	**5**	**6**	**7**	**8**	**9**	**10**

15. **Thinking about when you are in your bedroom/ living room/ home office with the windows closed, do you hear noise from service installations (lift, power generator, ventilation machinery) that come from communal areas in your building?** Yes/No

IF YES, GO TO 15a

15.a How much does noise from service installations (lift, power generator, ventilation machinery) that come from communal areas in your building annoy you?

This uses a scale from 0-to-10. If you are not annoyed at all choose 0; if you are extremely annoyed choose 10; otherwise, choose a number between 0 and 10, that represents your annoyance.

0 = Not at all annoyed										10 = Extremely annoyed
**0**	**1**	**2**	**3**	**4**	**5**	**6**	**7**	**8**	**9**	**10**

16. **Thinking about when you are in your bedroom/ living room/ home office with the windows closed, do you hear any other noise from communal areas in your building?** Yes/No

IF YES, GO TO 16a AND 16b. IF NO, GO TO 18

16.a Please describe this noise source here __________________

16.b How much does this noise from communal areas in your building annoy you?

This uses a scale from 0-to-10. If you are not annoyed at all choose 0; if you are extremely annoyed choose 10; otherwise, choose a number between 0 and 10, that represents your annoyance.

0 = Not at all annoyed										10 = Extremely annoyed
**0**	**1**	**2**	**3**	**4**	**5**	**6**	**7**	**8**	**9**	**10**

17. **Thinking about when you are in your bedroom/ living room/ home office with the windows closed, do you hear any other noise from communal areas in your building?** Yes/No

IF YES, GO TO 17a AND 17b. IF NO, GO TO 18

17.a Please describe this noise source here __________________

17.b How much does this noise from communal areas in your building annoy you?

This uses a scale from 0-to-10. If you are not annoyed at all choose 0; if you are extremely annoyed choose 10; otherwise, choose a number between 0 and 10, that represents your annoyance.

0 = Not at all annoyed										10 = Extremely annoyed
**0**	**1**	**2**	**3**	**4**	**5**	**6**	**7**	**8**	**9**	**10**

NOISE FROM NEIGHBOURS' PROPERTY*

18. **When you are in your bedroom/ living room/ home office with the windows closed, do you hear noise from the neighbours' property (e.g. talking, shouting, footsteps, jumping, running, dropped objects)?** Yes/No

IF YES, GO TO 19. IF NO, GO TO 36

19. **Thinking about when you are in your bedroom/ living room/ home office with the windows closed, do you hear noise from talking and/or shouting that come from your neighbours' property?** Yes/No

IF YES, GO TO 19a

19.a How much does noise from talking and/or shouting that come from your neighbours' property annoy you?

This uses a scale from 0-to-10. If you are not annoyed at all choose 0; if you are extremely annoyed choose 10; otherwise, choose a number between 0 and 10, that represents your annoyance.

0 = Not at all annoyed										10 = Extremely annoyed
**0**	**1**	**2**	**3**	**4**	**5**	**6**	**7**	**8**	**9**	**10**

20. **Thinking about when you are in your bedroom/ living room/ home office with the windows closed, do you hear noise from footsteps and/or jumping and/or running that come from your neighbours' property?** Yes/No

IF YES, GO TO 20a

20.a How much does noise from footsteps and/or jumping and/or running that come from your neighbours' property annoy you?

This uses a scale from 0-to-10. If you are not annoyed at all choose 0; if you are extremely annoyed choose 10; otherwise, choose a number between 0 and 10, that represents your annoyance.

0 = Not at all annoyed										10 = Extremely annoyed
**0**	**1**	**2**	**3**	**4**	**5**	**6**	**7**	**8**	**9**	**10**

21. **Thinking about when you are in your bedroom/ living room/ home office with the windows closed, do you hear noise from dropped objects (e.g. children dropping toys) that come from your neighbours' property?** Yes/No

<u>*IF YES, GO TO 21a*</u>

21.a How much does noise from dropped objects (e.g. children dropping toys) that come from your neighbours' property annoy you?

This uses a scale from 0-to-10. If you are not annoyed at all choose 0; if you are extremely annoyed choose 10; otherwise, choose a number between 0 and 10, that represents your annoyance.

0 = Not at all annoyed										10 = Extremely annoyed
**0**	**1**	**2**	**3**	**4**	**5**	**6**	**7**	**8**	**9**	**10**

22. **Thinking about when you are in your bedroom/ living room/ home office with the windows closed, do you hear noise from movement of furniture (e.g. chairs dragged across the floor) that come from your neighbours' property?** Yes/No

<u>*IF YES, GO TO 22a*</u>

22.a How much does noise from movement of furniture (e.g. chairs dragged across the floor) that come from your neighbours' property annoy you?

This uses a scale from 0-to-10. If you are not annoyed at all choose 0; if you are extremely annoyed choose 10; otherwise, choose a number between 0 and 10, that represents your annoyance.

0 = Not at all annoyed										10 = Extremely annoyed
**0**	**1**	**2**	**3**	**4**	**5**	**6**	**7**	**8**	**9**	**10**

23. **Thinking about when you are in your bedroom/ living room/ home office with the windows closed, do you hear noise from doors closing that come from your neighbours' property?** Yes/No

IF YES, GO TO 23a

23.a How much does noise from doors closing that come from your neighbours' property annoy you?

This uses a scale from 0-to-10. If you are not annoyed at all choose 0; if you are extremely annoyed choose 10; otherwise, choose a number between 0 and 10, that represents your annoyance.

0 = Not at all annoyed										10 = Extremely annoyed
**0**	**1**	**2**	**3**	**4**	**5**	**6**	**7**	**8**	**9**	**10**

24. **Thinking about when you are in your bedroom/ living room/ home office with the windows closed, do you hear noise from TV and/or Music and/or Video games and/or Radio that come from your neighbours' property?** Yes/No

IF YES, GO TO 24a

24.a How much does noise from TV and/or music and/or video games and/or radio that come from your neighbours' property annoy you?

This uses a scale from 0-to-10. If you are not annoyed at all choose 0; if you are extremely annoyed choose 10; otherwise, choose a number between 0 and 10, that represents your annoyance.

0 = Not at all annoyed										10 = Extremely annoyed
**0**	**1**	**2**	**3**	**4**	**5**	**6**	**7**	**8**	**9**	**10**

25. **Thinking about when you are in your bedroom/ living room/ home office with the windows closed, do you hear noise from telephone ringing that come from your neighbours' property?** Yes/No

IF YES, GO TO 25a

25.a How much does noise from telephone ringing that come from your neighbours' property annoy you?

This uses a scale from 0-to-10. If you are not annoyed at all choose 0; if you are extremely annoyed choose 10; otherwise, choose a number between 0 and 10, that represents your annoyance.

0 = Not at all annoyed										10 = Extremely annoyed
**0**	**1**	**2**	**3**	**4**	**5**	**6**	**7**	**8**	**9**	**10**

26. **Thinking about when you are in your bedroom/ living room/ home office with the windows closed, do you hear noise from light switches (turned on and off) and/or electric sockets (plugs being inserted into sockets) that come from your neighbours' property?** Yes/No

IF YES, GO TO 26a

26.a How much does noise from light switches (turned on and off) and/or electric sockets (plugs being inserted into sockets) that come from your neighbours' property annoy you?

This uses a scale from 0-to-10. If you are not annoyed at all choose 0; if you are extremely annoyed choose 10; otherwise, choose a number between 0 and 10, that represents your annoyance.

0 = Not at all annoyed										10 = Extremely annoyed
**0**	**1**	**2**	**3**	**4**	**5**	**6**	**7**	**8**	**9**	**10**

27. **Thinking about when you are in your bedroom/ living room/ home office with the windows closed, do you hear noise from home appliances (washing machine, dish washer, tumble dryer, vacuum cleaner) that come from your neighbours' property?** Yes/No

IF YES, GO TO 27a

27.a How much does noise from home appliances (washing machine, dish washer, tumble dryer, vacuum cleaner) that come from your neighbours' property annoy you?

This uses a scale from 0-to-10. If you are not annoyed at all choose 0; if you are extremely annoyed choose 10; otherwise, choose a number between 0 and 10, that represents your annoyance.

0 = Not at all annoyed										10 = Extremely annoyed
**0**	**1**	**2**	**3**	**4**	**5**	**6**	**7**	**8**	**9**	**10**

28. **Thinking about when you are in your bedroom/ living room/ home office with the windows closed, do you hear noise from dogs barking that come from your neighbours' property?** Yes/No

IF YES, GO TO 28a

28.a How much does noise from dogs barking that come from your neighbours' property annoy you?

This uses a scale from 0-to-10. If you are not annoyed at all choose 0; if you are extremely annoyed choose 10; otherwise, choose a number between 0 and 10, that represents your annoyance.

0 = Not at all annoyed										10 = Extremely annoyed
**0**	**1**	**2**	**3**	**4**	**5**	**6**	**7**	**8**	**9**	**10**

29. **Thinking about when you are in your bedroom/ living room/ home office with the windows closed, do you hear noise from water installations (water sounds from pipes, plumbing, flushing toilet, shower, bath) that come from your neighbours' property?** Yes/No

IF YES, GO TO 29a

29.a How much does noise from water installations (water sounds from pipes, plumbing, flushing toilet, shower, bath) that come from your neighbours' property annoy you?

This uses a scale from 0-to-10. If you are not annoyed at all choose 0; if you are extremely annoyed choose 10; otherwise, choose a number between 0 and 10, that represents your annoyance.

0 = Not at all annoyed										10 = Extremely annoyed
**0**	**1**	**2**	**3**	**4**	**5**	**6**	**7**	**8**	**9**	**10**

30. **Thinking about when you are in your bedroom/ living room/ home office with the windows closed, do you hear any other noise from your neighbours' property?** Yes/No

IF YES, GO TO 30a AND 30b. IF NO, GO TO 32

30.a Please describe this noise source here __________________

30.b How much does this noise from your neighbours' property annoy you?

This uses a scale from 0-to-10. If you are not annoyed at all choose 0; if you are extremely annoyed choose 10; otherwise, choose a number between 0 and 10, that represents your annoyance.

0 = Not at all annoyed										10 = Extremely annoyed
**0**	**1**	**2**	**3**	**4**	**5**	**6**	**7**	**8**	**9**	**10**

31. **Thinking about when you are in your bedroom/ living room/ home office with the windows closed, do you hear any other noise from your neighbours' property?** Yes/No

IF YES, GO TO 31a AND 31b. IT NO, GO TO 32

31.a Please describe this noise source here __________________

31.b How much does this noise from your neighbours' property annoy you?

This uses a scale from 0-to-10. If you are not annoyed at all choose 0; if you are extremely annoyed choose 10; otherwise, choose a number between 0 and 10, that represents your annoyance.

0 = Not at all annoyed		10 = Extremely annoyed

0	1	2	3	4	5	6	7	8	9	10

<u>RELATIONSHIP WITH YOUR NEIGHBOUR</u>

32. Do you know which of your neighbours causes the noise?

( ) None of them ( ) Some of them ( ) All of them ( ) I don't have noisy neighbours

<u>IF YOU CHOOSE SOME OR ALL:</u>

32a How would you describe your relationship with this neighbour that lives (answer as applicable):

a. Above you

( ) not at all close ( ) slightly close ( ) moderately close ( ) very close ( ) extremely close

b. Below you

( ) not at all close ( ) slightly close ( ) moderately close ( ) very close ( ) extremely close

c. On one side

( ) not at all close ( ) slightly close ( ) moderately close ( ) very close ( ) extremely close

d. On the other side

( ) not at all close ( ) slightly close ( ) moderately close ( ) very close ( ) extremely close

<u>SENSITIVITY TO NOISE</u>

33. Thinking about the noise you experience when you are in your home that comes from your neighbours' property, please indicate your agreement with the following statements.

This uses a 1-to-5 scale. If you disagree strongly choose 1; if you agree strongly choose 5; otherwise, choose a number between 2 or 4, that represents your level of agreement.

1. **I feel vulnerable when it is noisy and I am trying to get to sleep.**
   Disagree strongly 1 2 3 4 5 Agree strongly
2. **I don't feel happy when it is noisy.**
   Disagree strongly 1 2 3 4 5 Agree strongly
3. **I am easily awakened by noise.**
   Disagree strongly 1 2 3 4 5 Agree strongly
4. **It is difficult for me to adapt to noise**.

   Disagree strongly 1 2 3 4 5 Agree strongly
5. **I don't feel safe when it is noisy.**
   Disagree strongly 1 2 3 4 5 Agree strongly

6. **I find it difficult to get to sleep when there is noise.**
   Disagree strongly 1 2 3 4 5 Agree strongly
7. **I am sensitive to noise.**
   Disagree strongly 1 2 3 4 5 Agree strongly
8. **I cannot relax when it is noisy.**
   Disagree strongly 1 2 3 4 5 Agree strongly
9. **I cannot concentrate when it is noisy.**
   Disagree strongly 1 2 3 4 5 Agree strongly

<u>REACTION TO NOISE</u>

34. **Thinking about the noise you experience when you are in your home that comes from your neighbours' property, how often do you react as indicated below?**

This uses a 1-to-5 frequency scale. If your answer is never, choose 1; if it is always choose 5; otherwise, choose a number between 1 to 5, that represents how frequently it happens to you

a. **I accept that I cannot do anything to stop the noise.**

   Never 1 2 3 4 5 Always

b. **I talk to someone about the problem.**

   Never 1 2 3 4 5 Always

c. **I start to talk louder when I'm in a conversation.**

   Never 1 2 3 4 5 Always

d. **I increase the volume of the radio/TV/music.**

   Never 1 2 3 4 5 Always

e. **I think about moving to a different property.**

   Never 1 2 3 4 5 Always

f. **I go outside.**

   Never 1 2 3 4 5 Always

g. **I imagine (or daydream about) a better time or place.**

   Never 1 2 3 4 5 Always

h. **I outwardly express anger towards the neighbours that create the noise.**

   Never 1 2 3 4 5 Always

i. **I resign myself to it.**

   Never 1 2 3 4 5 Always

j. **I use an activity to distract me.**

   Never 1 2 3 4 5 Always

k. **I knock on the wall, floor or ceiling or shout.**

Never 1 2 3 4 5 Always

l. I inwardly express anger towards the neighbours that create the noise.

Never 1 2 3 4 5 Always

m. I go into a different room.

Never 1 2 3 4 5 Always

n. I say to my neighbour that I don't hear them, even though I do.

Never 1 2 3 4 5 Always

o. I try to ignore the noise.

Never 1 2 3 4 5 Always

COMPLAINTS

35. Have you ever complained about the noise from your neighbours' property?
( ) Yes ( ) No

IF YES:

35.a. How did you complain? You may select more than one.

a. By calling the neighbour on the intercom or by telephone
b. By writing a note to the neighbour
c. By complaining directly to the neighbour (Face to face contact)
d. By bringing up the question of "noise" at meetings of the tenants
e. By contacting the property management office
f. By calling the police and/or local security
g. By contacting a Government office or/and local council (e.g. __________)
h. Other:

DEMOGRAPHIC DATA

36. Age: ___18 to 35 ___36 to 50 ___51 to 64 ___ 65 or over

37. Gender: ___Male ___Female

38. Are you British/Brazilian/Korean? (DELETE AS APPLICABLE)
( )Yes ( )No____________ *(IF NO, SPECIFY YOUR NATIONALITY)*

39. Education

a. School level
b. University level

40. Employment Status

a. Full-Time worker
b. Part-Time worker
c. Self-employed
d. Unemployed
e. Student
f. Homemaker (looking after family at home)
g. Retired

<u>*IF YOU CHOSE a, b or c, GO TO 39a*</u>

39.a What are your work hours?

a. Day shift
b. Night shift
c. Varies between night and day shifts

41. **Annual household income (VALUES WILL BE CONVERTED TO CURRENCIES FOR KOREA AND BRAZIL)**

a. Below £10,700 per annum
b. Between £10,700 and £53,500 per annum
c. Between £53, 500 and £107,700 per annum
d. At least £107,700 per annum
e. Not known

42. People you live with

No-one (i.e. alone)
With people who are not your family
With family including children under the age of 12 years old
With family including children over the age of 12 years old
With family but without children

***Thank you for participating in our study about attitudes to noise inside dwellings in London/Seoul/Sao Paulo.* (DELETE AS APPLICABLE)**
